# Müller glia-derived PRSS56 is required to sustain ocular axial growth and prevent refractive error

**DOI:** 10.1371/journal.pgen.1007244

**Published:** 2018-03-12

**Authors:** Seyyedhassan Paylakhi, Cassandre Labelle-Dumais, Nicholas G Tolman, Michael A. Sellarole, Yusef Seymens, Joseph Saunders, Hesham Lakosha, Wilhelmine N. deVries, Andrew C. Orr, Piotr Topilko, Simon WM. John, K. Saidas Nair

**Affiliations:** 1 Department of Ophthalmology, University of California, San Francisco, California, United States of America; 2 Howard Hughes Medical Institute, The Jackson Laboratory, Bar Harbor, ME, United States of America; 3 Department of Ophthalmology and Visual Sciences, Dalhousie University, Halifax, NS, Canada; 4 Ecole Normale Supérieure, Institut de Biologie de l’ENS (IBENS), and Inserm U1024, and CNRS UMR 8197, Paris, France; 5 Department of Ophthalmology, Tufts University School of Medicine Boston, MA, United States of America; 6 Department of Anatomy, University of California, San Francisco, California, United States of America; Stanford University School of Medicine, UNITED STATES

## Abstract

A mismatch between optical power and ocular axial length results in refractive errors. Uncorrected refractive errors constitute the most common cause of vision loss and second leading cause of blindness worldwide. Although the retina is known to play a critical role in regulating ocular growth and refractive development, the precise factors and mechanisms involved are poorly defined. We have previously identified a role for the secreted serine protease PRSS56 in ocular size determination and *PRSS56* variants have been implicated in the etiology of both hyperopia and myopia, highlighting its importance in refractive development. Here, we use a combination of genetic mouse models to demonstrate that *Prss56* mutations leading to reduced ocular size and hyperopia act via a loss of function mechanism. Using a conditional gene targeting strategy, we show that PRSS56 derived from Müller glia contributes to ocular growth, implicating a new retinal cell type in ocular size determination. Importantly, we demonstrate that persistent activity of PRSS56 is required during distinct developmental stages spanning the pre- and post-eye opening periods to ensure optimal ocular growth. Thus, our mouse data provide evidence for the existence of a molecule contributing to both the prenatal and postnatal stages of human ocular growth. Finally, we demonstrate that genetic inactivation of *Prss56* rescues axial elongation in a mouse model of myopia caused by a null mutation in *Egr1*. Overall, our findings identify PRSS56 as a potential therapeutic target for modulating ocular growth aimed at preventing or slowing down myopia, which is reaching epidemic proportions.

## Author summary

Refractive errors mainly occur when changes in ocular size (ocular axial length) prevent light from focusing directly on the retina. Myopia (nearsightedness) is the most common form of refractive errors in which the focused image falls in front of the retina. The recent unprecedented rise in the incidence of myopia has significant implications as individuals with high myopia are at an increased risk of developing irreversible blinding conditions, including retinal detachment, macular degeneration, and glaucoma. Ocular axial growth is a key determinant of normal refractive development. Although the retina has been established as a central player involved in the regulation of ocular growth, the specific retinal cell type(s) and molecular pathways involved are poorly defined. Here, we have utilized genetic mouse models to provide significant insight into spatial and temporal requirements of the retinal factor PRSS56 in ocular size determination. Importantly, we have uncovered a previously unrecognized role for retinal Müller glia in ocular growth and demonstrated that *Prss56* inactivation has translational potential to rescue axial length elongation in a mouse model of myopia. Collectively, our findings suggest that therapeutic strategies targeting PRSS56 to modulate ocular growth could have important clinical implications to prevent or slowdown the progression of myopia and associated blinding conditions in humans.

## Introduction

A central feature of organogenesis is the intrinsic ability to faithfully determine final organ size and shape. This is particularly important in the case of the eye where size determination is a complex and tightly coordinated process critical for achieving optimal vision. During ocular refractive development, precise ocular growth regulation is essential to ensure that the eye’s axial length matches the optical focal plane, enabling focused images to fall on the retina (emmetropization), resulting in clear vision. Alterations in ocular axial length constitute the major cause of refractive errors. Shortened or elongated axial length result in focused images falling behind or in front of the retina leading to hyperopia and myopia, respectively [1, 2].

Uncorrected refractive error is the most common cause of vision loss, and the second leading cause of blindness after cataract [3]. Notably, the prevalence of myopia, the most common form of refractive errors, is rising rapidly, reaching epidemic proportions in some countries [4–6]. This has significant public health implications as individuals with high myopia are at disproportionately increased risk of developing irreversible blinding conditions including retinal detachment, myopic macular degeneration, cataract, and glaucoma [7]. Thus, there is an urgent need to identify interventions offering the promise of modulating ocular growth, restoring healthy refractive development, and preventing associated blinding conditions.

Although environmental factors have a strong influence in determining an individual’s refractive status, genetic factors account for over 50% of the variability in refractive status within populations [8–10]. Notably, mutations in genes involved in ocular size determination have been implicated in a subset of refractive errors with a strong developmental basis (high myopia and high hyperopia/nanophthalmos) [11–14]. More common forms of myopia are thought to have a complex multifactorial etiology resulting from an intricate interplay between multiple genetic and environmental factors [8, 9], and genome-wide association studies (GWAS) have led to the identification of several genes/loci linked to myopia as well as those accounting for natural variations in refraction in the general population [15, 16]. An improved understanding of the contribution of individual genes, the pathways involved and their interactions will provide the necessary framework to define the mechanisms underlying refractive development and associated errors, and identify potential targets for therapeutic intervention [5].

Ocular growth broadly comprises two distinct phases. In humans, the first phase occurs prenatally and is primarily dictated by genetic factors [14], while the second phase takes place postnatally and relies on a complex interplay between environmental and genetic factors [17]. Notably, postnatal ocular growth takes place when the eyes are developmentally open and is highly dependent on visual experience, which modulates the rate of ocular growth. Consistent with a requirement for patterned visual stimulation in ocular size determination, studies in animal models have suggested an important role for retinal neurotransmitters and neuromodulators in postnatal ocular growth and normal refractive development [18–20]. Signals emanating from the retina are thought to be relayed to the sclera to promote remodeling of the scleral extracellular matrix (ECM), a key step necessary to support ocular axial growth [21].

The use of genetically tractable animal models such as the mouse has recently gained popularity to study ocular size determination and emmetropization [22]. The mouse eye responds to form deprivation and lens defocus suggesting the existence of a functional emmetropization mechanism similar to that operating in humans [22–24], and recent technological advances have allowed precise measurement of refraction and ocular size in mice [25–27]. Importantly, studies in the mouse have guided the identification and validation of genes, pathways and mechanisms involved in ocular growth, refractive development and associated errors [22, 28–35]. For instance, mice with a null mutation of the transcriptional factor *Egr1* recapitulate the ocular axial length elongation and refractive shift characteristic of myopia and constitute a useful animal model to dissect the mechanisms involved in ocular growth and myopia [31]. In addition, we have previously demonstrated that mice carrying a mutation in the gene coding for the secreted trypsin-like serine protease PRSS56 *(Prss56*^*glcr4*^) exhibit reduced ocular size [36]. Mice homozygous for the *Prss56*^*glcr4*^ mutation have reduced ocular axial length, primarily caused by a decrease in posterior segment size, without any gross morphological changes in ocular tissues [36]. Furthermore, we and other groups have shown that *PRSS56* mutations lead to nanophthalmos (posterior microphthalmia) and extreme hyperopia characterized by significant reduction in ocular axial length in humans [36–39]. Interestingly, GWAS from multiple independent groups found an association between *PRSS56* and myopia, suggesting that common *PRSS56* variants may also participate in the manifestation of more complex and common forms of refractive errors [16, 40]. The implication of *PRSS56* variants in both hyperopia and myopia suggests a critical role for PRSS56 in ocular axial growth regulation and refractive development. The availability of *Prss56* mutant mice exhibiting reduced ocular axial length and recapitulating hallmark features of human nanophthalmos constitutes a unique resource to mechanistically dissect the molecular pathways contributing to ocular size determination.

Here we have used a combination of genetic mouse models to perform a detailed molecular and cellular characterization of the role of PRSS56 in ocular size determination. Our results establish a previously unknown role of Müller glia in the regulation of ocular axial growth and refractive development. Significantly, we demonstrate a requirement for PRSS56 activity to support ocular growth during distinct developmental stages spanning the pre- and post-eye opening periods, suggesting that at least some molecules contribute to both the prenatal and postnatal stages of ocular growth in humans. Importantly, we demonstrate that genetic inactivation of *Prss56* can rescue axial elongation in a mouse model of myopia caused by loss of EGR1 function.

### Results

#### Loss of PRSS56 function contributes to ocular axial length reduction and hyperopia

The *Prss56* mutations we have previously identified in mice (ENU- induced mutation, *Prss56*^*glcr4*^) and humans (c.1059_1066insC, p.Gln356Pro fsX152) give rise to a truncated protein lacking the C-terminal region, leaving the catalytic domain intact [36]. In agreement with the presence of a functional catalytic domain, we have previously demonstrated intact trypsin-like serine protease activity of the recombinant PRSS56 mutant protein [36]. Similarly, human *PRSS56* mutations identified by independent groups are not predicted to completely disrupt PRSS56 catalytic activity [37–39]. Thus, it is currently unknown whether *PRSS56* mutations causing ocular size reduction act via a loss or gain of function mechanism.

**Fig 1:**
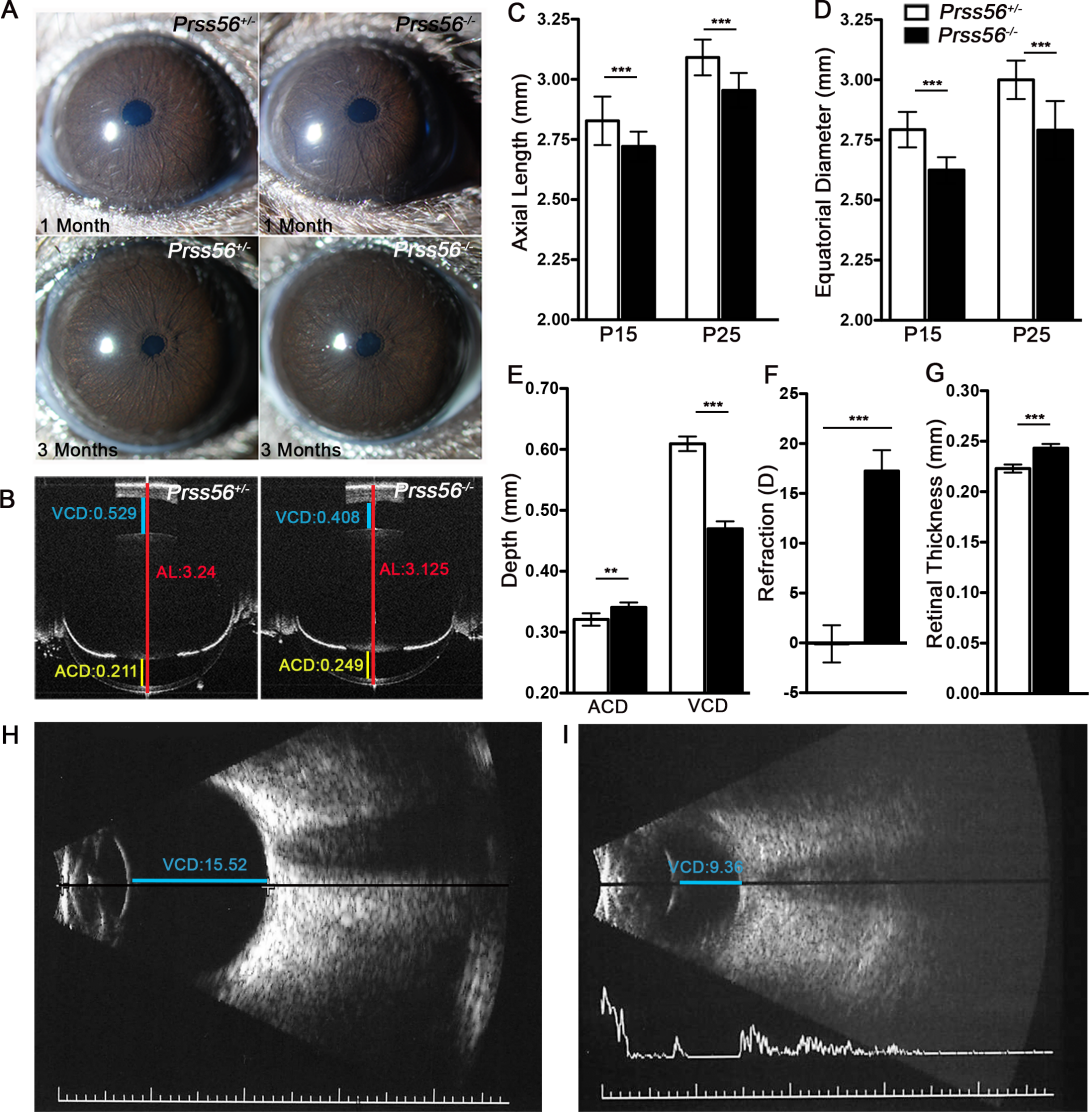
*Prss56*^-/-^ eyes exhibit reduced ocular axial length and hyperopia. (**A**) Representative images of slit-lamp examination by broad-beam illumination to assess ocular structures including the iris, pupil, and lens at 1 and 3 months of age. *Prss56*^*-/-*^ eyes did not exhibit any obvious structural abnormalities and were indistinguishable from *Prss56*^*+/-*^ eyes. (**B**) Representative OCT images demonstrating a reduction in ocular size in *Prss56*^*-/-*^ compared to *Prss56*^*+/-*^ mice (shown are P30 eyes). The red, blue and yellow lines indicate ocular axial length (AL), vitreous chamber depth (VCD) and anterior chamber depth (ACD), respectively. (**C**-**D**) *Prss56*^*-/-*^ eyes exhibit a modest but highly significant reduction in axial length (**C**) and equatorial diameter (**D**) at P15 and P25. (**E**) A significant reduction in VCD and increase in ACD was detected in *Prss56*^*-/-*^ compared to *Prss56*^+/-^ eyes (shown are data from P30 eyes). (**F**) Consistent with reduced ocular size, *Prss56*^*-/-*^ mice display a hyperopic refraction compared to *Prss56*^*+/-*^ littermates (shown are data from 2-months old mice). (**G**) Retinal thickness was significantly increased in *Prss56*^*-/-*^ compared to *Prss56*^*+/-*^ eyes. Values are presented as mean ± SD, *** p<0.001, t-test. **C** and **D:** N > 10 per group; **E** and **F:** N ≥ 6 per group; and **G:** N≥ 4. (**H**-**I**) Representative B-scan images of eyes from an unaffected individual (**H**) and an individual with a *PRSS56* mutation (**I**). VCD is substantially reduced in the eye of the individual carrying a mutant *PRSS56* allele compared to a normal emmetropic eye.

To determine if the reduction in ocular size observed in *Prss56* mutant mice *(Prss56*^*glcr4/glcr4*^) results from loss of PRSS56 function, we characterized the ocular phenotypes of a mouse strain carrying a null allele of *Prss56 (Prss56*^*Cre*^ also referred to as *Prss56*^*-*^ in the manuscript). This strain was generated by replacing exon1 of *Prss56* with a sequence coding for CRE recombinase resulting in a null allele and *Prss56* promoter-driven CRE expression [41]. As the ocular size of mice heterozygous for the *Prss56*^*Cre*^ allele (*Prss56*^*+/-*^) was indistinguishable from that of their wild-type littermates (S1A and S1B Fig), *Prss56*^*+/-*^ mice were used as controls for most of our experiments unless otherwise specified. Slit-lamp eye examination did not reveal any difference between mice homozygous for the null allele (*Prss56*^*-/-*^*)* and control mice (Fig 1A). Next, we performed a detailed ocular biometric analysis to assess various parameters, including axial length, equatorial diameter, vitreous chamber depth (VCD), anterior chamber depth (ACD), and lens thickness (as seen in OCT images, Fig 1B). Biometric analysis revealed reduced ocular size in *Prss56*^*-/-*^ mice, in which both ocular axial length and equatorial diameter were significantly smaller compared to their *Prss56*^*+/-*^ littermates at all ages examined (postnatal day (P) 15, P25 and P60) (Fig 1B–1D, S1C-S1G Fig). The VCD of *Prss56*^*-/-*^ eyes was significantly smaller compared to their *Prss56*^*+/-*^ littermates (Fig 1E). Interestingly, the ACD was marginally larger in *Prss56*^*-/-*^ compared to control eyes (Fig 1E), and no significant difference was observed in lens thickness (S1H Fig). These findings suggest that the reduction in post-equatorial segment primarily accounts for the decreased ocular axial length observed in *Prss56*^*-/-*^ mice. Consistent with their reduced ocular axial length, *Prss56*^*-/-*^ mice also exhibited hyperopic refraction (Fig 1F). In addition, retinal thickness was increased in *Prss56*^*-/-*^ eyes compared to control eyes (Figs 1G and S1F). A detailed summary of ocular biometric measurements is presented in S1 Table. Together these findings show that *Prss56*^*-/-*^ mice have reduced ocular size, develop hyperopia, and recapitulate the ocular phenotypes observed in the previously characterized ENU-induced *Prss56*^*glcr4*^ mutant mice [36], demonstrating that loss of PRSS56 function leads to reduced ocular size. We next performed the ocular biometry (A- and B-scan) on an individual exhibiting nanophthalmos caused by a homozygous *PRSS56* mutation (missense variant, p.G320R) [38]. Consistent with ocular findings in *Prss56*^*-/-*^ mice, the human *PRSS56* mutation led to a substantial reduction in VCD compared to a normal emmetropic eye (Fig 1H and 1I and Table 1), suggesting that *PRSS56* mutations leading to nanophthalmos and extreme hyperopia in humans likely act via a loss of function mechanism.

**Table 1:** Ocular biometry of an individual carrying a *PRSS56* mutation.

	Affected Right (mm)	Affected Left (mm)	Representative Normal (mm)
**Anterior Chamber Depth (ACD)**	**1.76**	**1.53**	**2.82**
**Lens Thickness**	**4.20**	**4.30**	**4.04**
**Vitreous Cavity Depth (VCD)**	**9.36**	**9.64**	**15.52**
**Axial Length**	**15.32**	**15.47**	**22.18**

#### *Prss56* ocular expression is restricted to the neural retina and is first detected in a pool of late retinal progenitor cells

**Fig 2:**
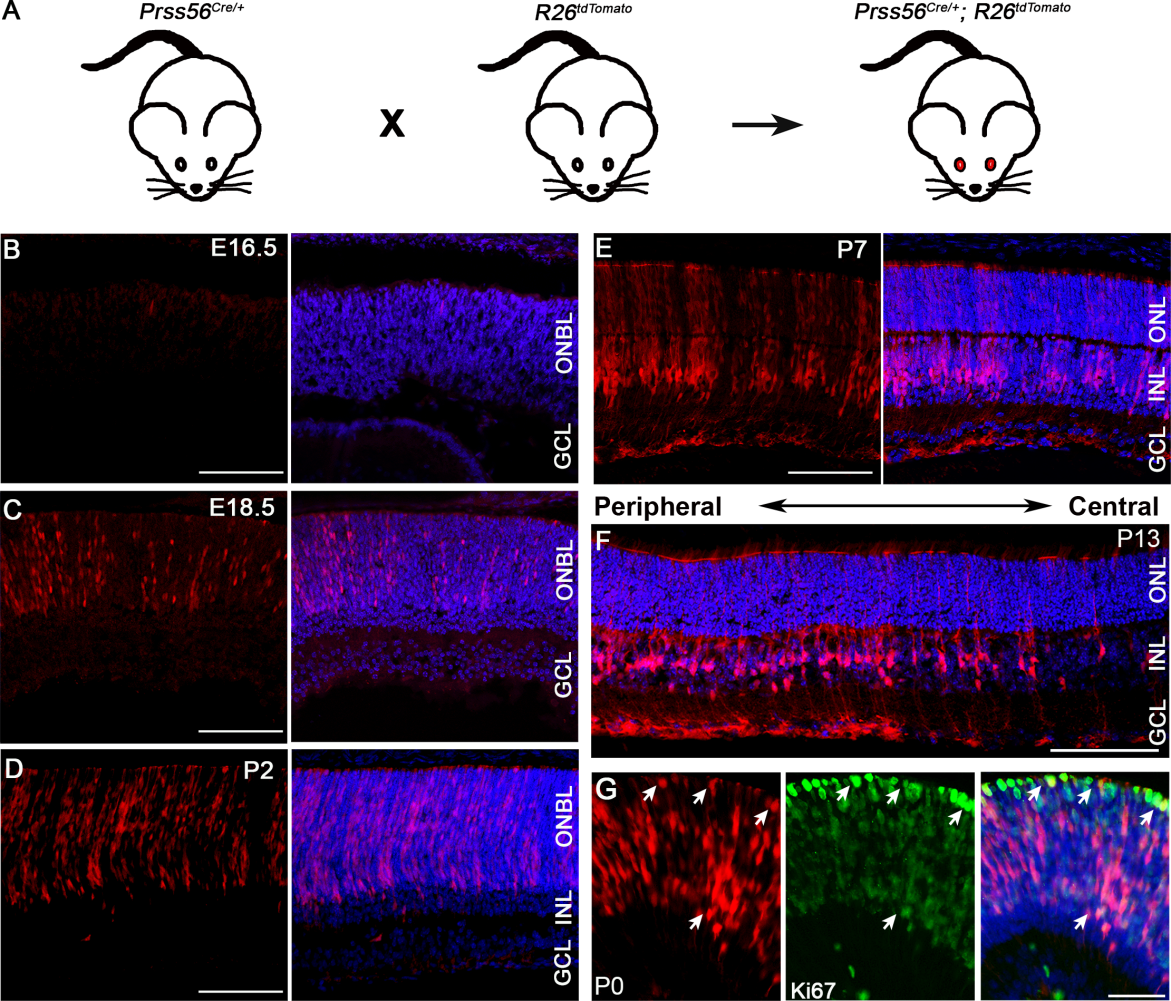
Lineage tracing of *Prss56* expressing cells during ocular development. **(A)** *Prss56*^*Cre/+*^ mice were crossed to *R26*^*tdTomato/+*^ reporter mice that express tdTomato following CRE-mediated excision of a stop codon to label *Prss56* expressing cells and their derivatives. (**B**-**F)** Representative images showing lineage tracing of *Prss56* expressing cells (red) in *Prss56*^*Cre/+*^; *R26*^*tdTomato/+*^ eyes throughout ocular development. (**B**) tdTomato expression is first detected in the retina at embryonic day (E) 16.5 in retinal progenitor cells (RPCs). (**C**, **D**) The number of tdTomato positive RPCs increases with age, shown are (**C**) E18.5 and (**D**) P2 retinas. (**E**) By P7, when retinal laminar organization is visible, tdTomato expression was predominantly observed in cells exhibiting characteristic features of Müller glia, with cell bodies located in the inner nuclear layer and apicobasal processes extending across the retina. tdTomato expression was also detected in the inner segment of rod photoreceptors. (**F**) tdTomato expression continues to be detected in Müller cells and rod photoreceptors following complete maturation of retinal cell types at P13. Interestingly, tdTomato-labeled cells were enriched in the peripheral region and relatively sparser in the central region of the retina. (**G**) Ki67 immunolabeling of P0 *Prss56*^*Cre/+*^; *R26*^*tdTomato/+*^ eyes demonstrate Ki67 expression in tdTomato positive retinal cells. E, embryonic day; GCL, ganglionic cell layer; INL, inner nuclear layer; ONBL, outer neuroblastic layer; ONL, outer nuclear layer; P, postnatal day. Scale bars: 100μm (**B**-**F**) and 50μm (**G**).

As a first step in addressing the role of PRSS56 in ocular size determination, we performed a lineage tracing experiment to determine the identity and fate of cells expressing *Prss56* in the developing eye. To this end, *Prss56*^*Cre/+*^ mice expressing CRE recombinase under the control of the *Prss56* promoter were bred to the inducible *R26*^*tdTomato*^ reporter mice that express *tdTomato* in presence of CRE (Fig 2A). In the resulting offspring, the *tdTomato* reporter gene will be expressed in *Prss56*-expressing cells and their derivatives, thereby allowing lineage tracing. Assessment of tdTomato fluorescence on ocular sections revealed that ocular *Prss56* expression is restricted to the retina during both embryonic and postnatal ages. *Prss56* expression is first detected in sparse cells in the outer neuroblastic layer (ONBL) at embryonic day (E) 16.5 (Fig 2B). The number of tdTomato positive retinal cells increases over time as shown at E18.5 and P2 (Fig 2C and 2D). As the retina matures and cells differentiate, intense tdTomato expression is detected in the inner nuclear layer (INL), and weaker signal in the photoreceptor layer (Fig 2E and 2F). Of note, tdTomato-positive cells are enriched in the peripheral retina relative to the central retina (Figs 2F, S2A and S2C) and no tdTomato signal was detected in other ocular tissues, including the sclera, choroid, cornea, lens, ciliary body and iridocorneal angle (S2C and S2D Fig).

**Fig 3:**
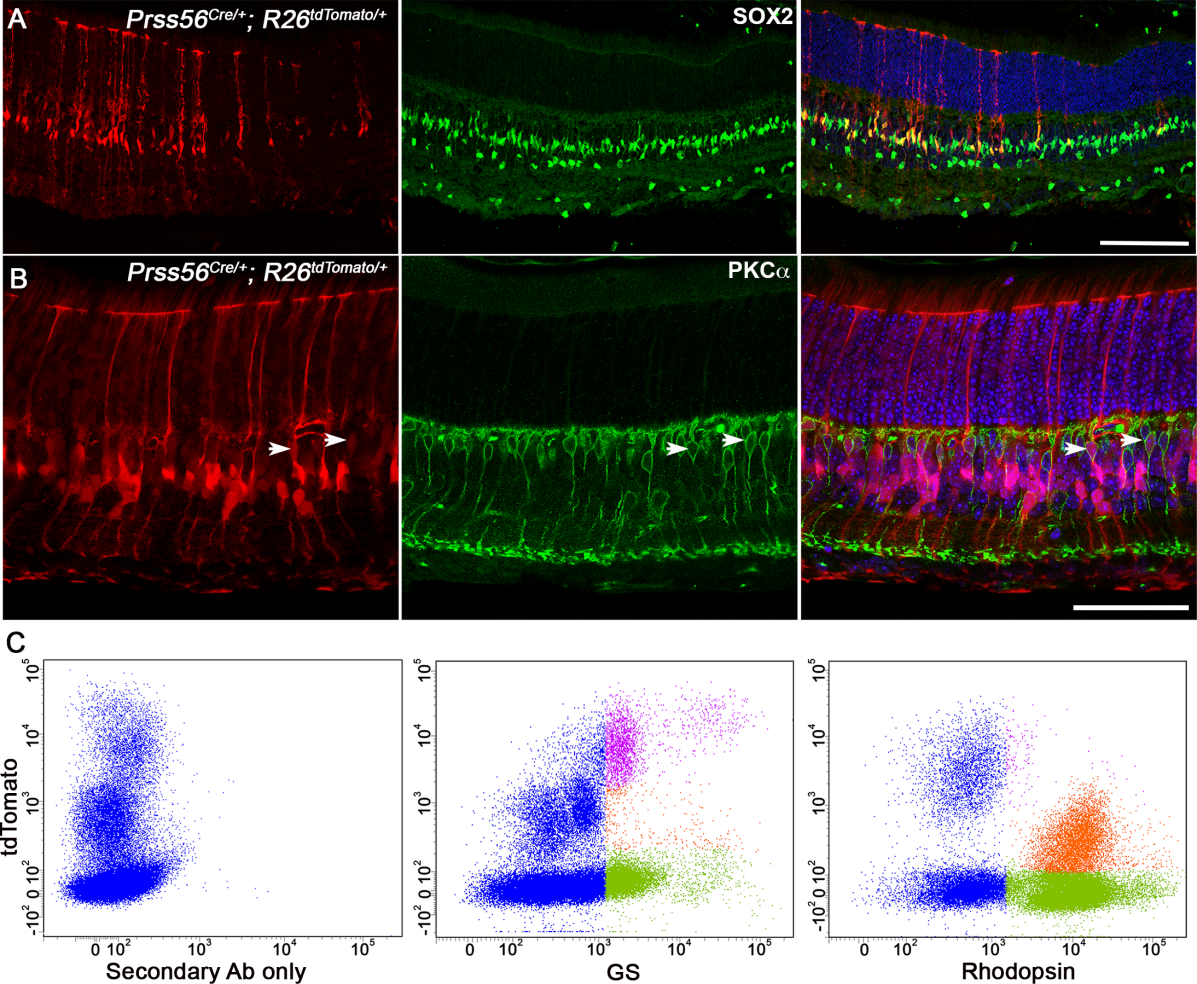
Earliest *Prss56* expression occurs in late retinal progenitor cells. (**A**, **B**) Representative images of *Prss56*^*Cre/+*^; *R26*^*tdTomato/+*^ retina immunolabeled for SOX2 (**A**) or PKCα (**B**). (**A**) tdTomato expression (red) is present in SOX2 immunopositive Müller cells (green). The peripheral and central regions of the retina are oriented left to right. (**B**) Representative images showing low tdTomato expression in a subset of PKCα immunolabeled bipolar cells in *Prss56*^*Cre/+*^; *R26*^*TdTomato/+*^ retina (arrows). (**C**) Flow cytometry analysis of Glutamine Synthetase (GS) and Rhodopsin expression in *Prss56*^*Cre/+*^; *R26*^*tdTomato/+*^ retinal cell suspensions. GS expression was predominantly detected in tdTomato negative (green) and high tdTomato expressing cells (purple). Rhodopsin expression was predominantly detected in tdTomato negative (green) and low tdTomato expressing (orange) cells. A minimum of 4 eyes per group was pooled for each retinal cell suspension and flow cytometry analyses were repeated 2–3 times on independent samples. Gating was established based on *Prss56*^*Cre/+*^; *R26*^*tdTomato/+*^ retinal cell suspension incubated with AlexaFluor 488 conjugated secondary antibody only. Together, these data demonstrate that *Prss56* is expressed by late RPCs that give rise to bipolar cells, rod photoreceptors, and Müller cells. Scale bars = 100μm(**A**) and 50μm (**B**).

The spatio-temporal pattern of tdTomato labeling in the retina is suggestive of *Prss56* being expressed in late retinal progenitor cells (RPCs) and its derivatives. To confirm this, we performed a series of immunolabeling studies. Supporting expression of *Prss56* by RPCs, we show that tdTomato positive cells express Ki67, a marker of dividing cells, in P0 retina (Fig 2G). In addition, we found that tdTomato positive cells located in the INL express the Müller glia markers vimentin and SOX2 (Figs S3A and 3A), as well as PKCα, a marker of bipolar cells (Fig 3B), demonstrating that tdTomato also labels late RPC derivatives. The reporter line used in our experiments showed an inherent variability in tdTomato fluorescence intensity between various retinal cell types. While intense tdTomato signal was detected in SOX2 and vimentin immunopositive Müller cells, weaker tdTomato signal was observed in PKCα immunopositive bipolar cells (Fig 3B, arrow). Using flow cytometry, we further demonstrate that Müller glial cells (immunolabeled for glutamate synthetase (GS)) exhibit more intense tdTomato signal, while rod photoreceptors (immunolabeled for Rhodopsin) exhibit weaker tdTomato signal (Fig 3C). Together, these findings demonstrate that *Prss56* is expressed in late RPCs fated to give rise to subsets of rod photoreceptor, bipolar and Müller cells.

#### *Prss56* expression is restricted to a subset of Müller glia following retinal cell differentiation

**Fig 4:**
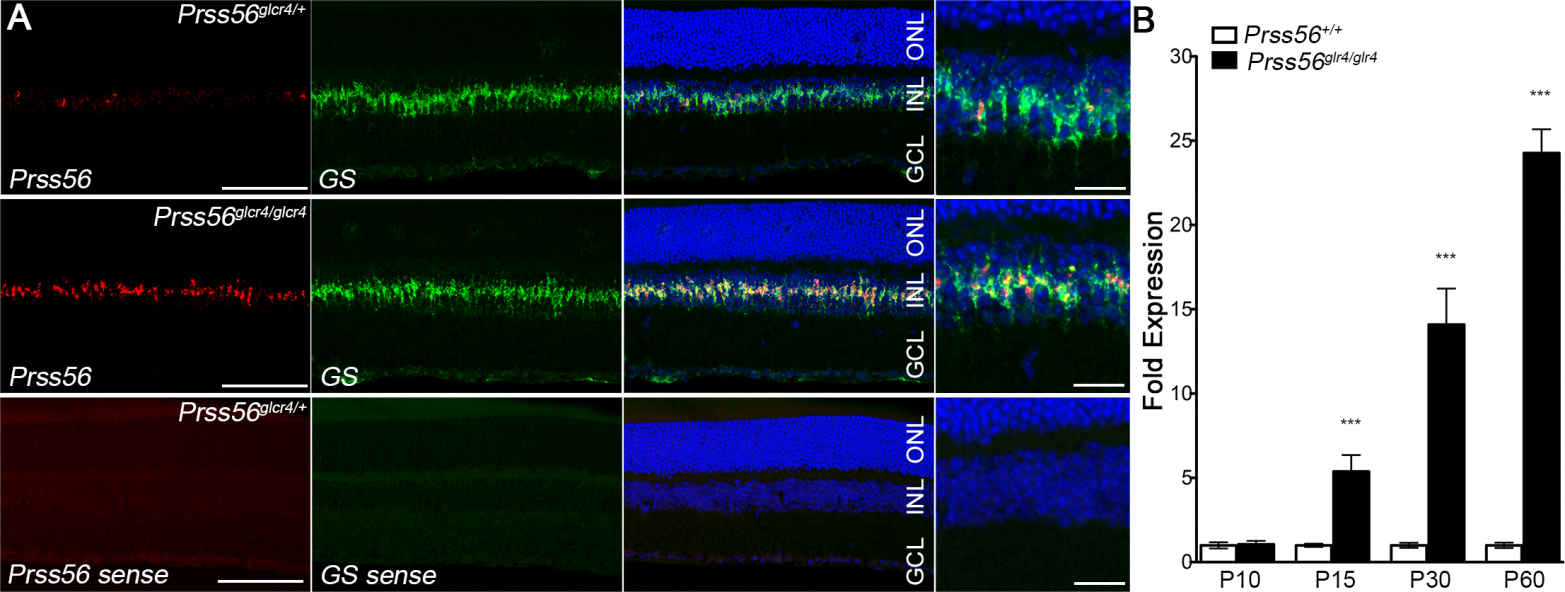
*Prss56* is predominantly expressed by a subset of Müller cells. (**A**) Dual fluorescent *in situ* hybridization for *Prss56 (red)* and *Glutamine Synthetase* (GS, green) showed localization of *Prss56* expression in the inner nuclear layer of the retina from adult *Prss56*^*glcr4/+*^ and *Prss56*^*glcr4/glcr4*^ mice (2 months old). *Prss56* expression colocalized with that of GS, a marker of Müller cells. Both the signal intensity and number of Müller glia expressing *Prss56* were substantially higher in *Prss56*^*glcr4/glcr4*^ compared to *Prss56*^*glcr4/+*^ retina, indicating increased *Prss56* expression in *Prss56* mutant retina. (**B**) Graph showing relative expression of *Prss56* mRNA levels using qPCR in wild-type and mutant retina at different developmental stages. Increased *Prss56* expression was detected in the mutant retina from P15 onward. *Prss56* expression was normalized to the expression of three housekeeping genes (*Hprt1*, *Actb1* and *Mapk1*). Data are presented as fold expression relative to wild-type (mean ± SEM), N≥4 /group. *** p<0.001, t-test. Scale bars= 100μm and 50μm for low and high magnification images in **A**.

The reporter (tdTomato) is not only expressed in cells actively transcribing *Prss56* but also in cells derived from parent cells expressing *Prss56*. Therefore, we performed *in situ* hybridization to determine the expression pattern of *Prss56* at critical time points during and following complete maturation of retinal cells. We show that *Prss56* expression is selectively detected in the INL of the retina during (P10) and following (P15) maturation of retinal cell types, and that *Prss56* expression is enriched in the peripheral retina relative to the central retina (S3B and S3C Fig). Dual immunofluorescent labeling using antisense probes for *GS* (glial cell marker) and *Prss56*, revealed colocalization of *Prss56 (red)* and *GS (green)* expression in the INL of adult retina, while no signal was detected using the sense probes (Fig 4A). Interestingly, *in situ* hybridization revealed a substantial increase in the number of *Prss56* expressing cells in *Prss56* mutant retina (*Prss56*^*glcr4/glcr4*^, ENU- induced mutation with C-terminal truncation) compared to control retina (*Prss56*^*glcr4*/+^), suggesting increased *Prss56* expression in mutant retina (Fig 4A). Quantitative PCR analysis further demonstrated a significant upregulation in the levels of *Prss56* mRNA in mutant retina (*Prss56*^*glcr4/glcr4*^) compared to their control counterparts from P15 onwards (Fig 4B). Collectively, these findings demonstrate that *Prss56* is selectively expressed by a subset of Müller glia following retinal maturation, and suggest the existence of a feedback regulatory loop modulating retinal *Prss56* expression in response to alteration in ocular size.

#### Early requirement for PRSS56 during ocular axial growth

**Fig 5:**
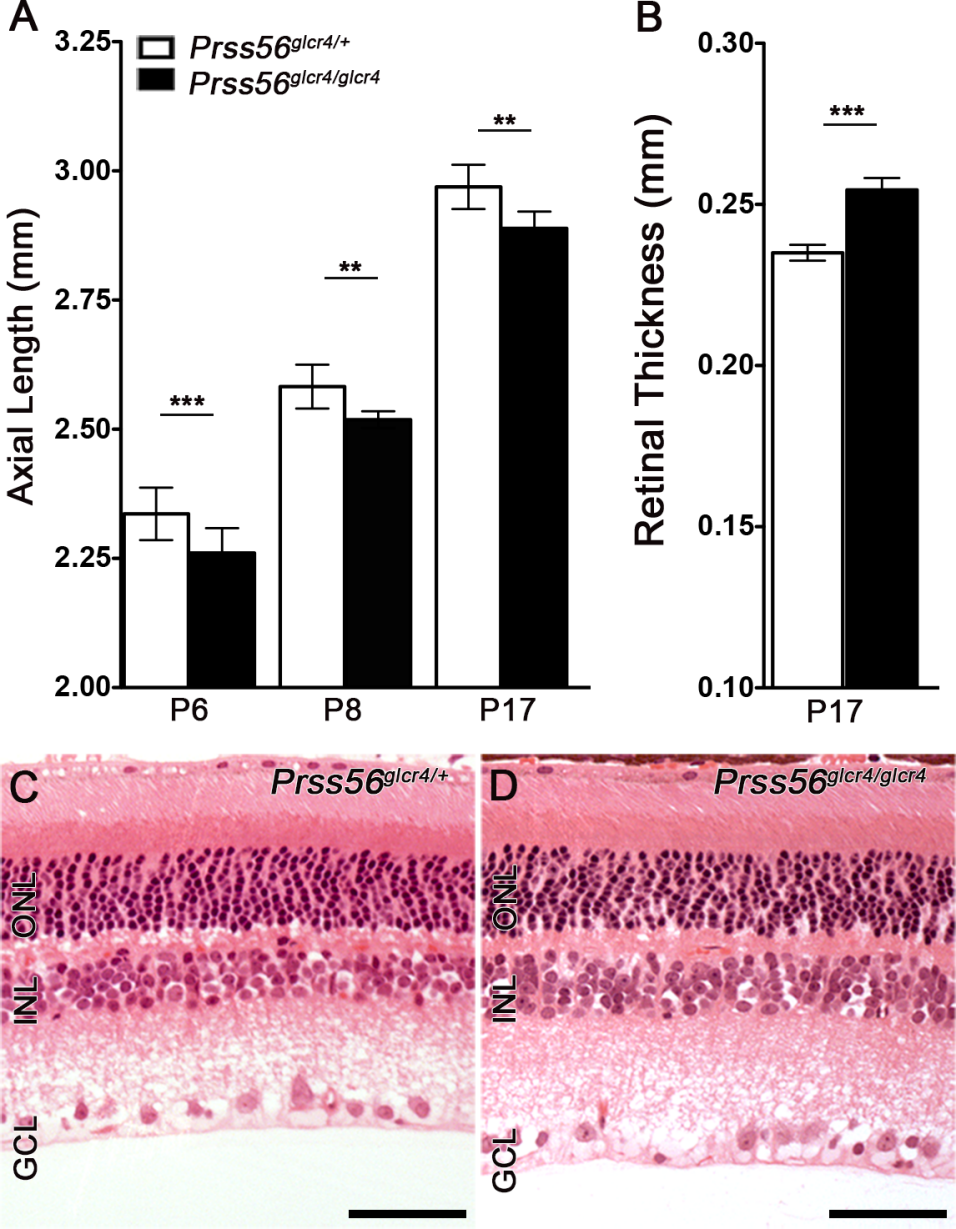
Early requirement for PRSS56 in ocular size determination. (**A**, **B**) Ocular biometric analysis by OCT revealed reduced ocular axial length (**A**) and increased retinal thickness (**B**) in *Prss56*^*glcr4/glcr4*^ eyes compared to *Prss56*^*glcr4/+*^ eyes at distinct developmental time points ranging from P6 to P17. Although both mutant and control mice exhibit an age-dependent increase in ocular size, ocular axial length is significantly reduced in *Prss56* mutant mice compared to control mice. The reduction in ocular axial length was detected as early as P6 (**A**). (**B**) OCT analysis revealed that *Prss56* mutant retina is significantly thicker than control retina (shown is P17). (**C**-**D**) Histological analysis revealed that the number of nuclear stacks in both the inner and outer retinal nuclear layers (INL and ONL, respectively) was consistently greater in the *Prss56* mutant retina (**D**) compared to control retina (**C**). For comparison between *Prss56* mutant and control mice: *p<0.05, ** p<0.01, *** p<0.001, t-test. Scale bars = 100μm. Values are presented as mean ± SD. N ≥ 12 per group for P6 measurements and N > 6 for P8 and P17 (**A**, **B**).

Having demonstrated a role for PRSS56 in ocular growth and its expression pattern during retinal development, we next assessed the temporal requirement for PRSS56 activity during ocular size determination. Using spectral-domain optical coherence tomography (SD-OCT), we detected a slight but significant reduction in axial length in *Prss56* mutant eyes compared to control eyes as early as P6 (Fig 5A). Moreover, *Prss56* mutant retina was significantly thicker than control retina at P17 and 2 months (Figs 5B and S1F and S1 Table). Histological analysis of *Prss56* mutant retina revealed no overt morphological defect with the exception of an increased number of nuclear stacks in both the outer and inner nuclear layers, which could contribute to the observed increase in retinal thickness (Fig 5C and 5D).

#### PRSS56 derived from Müller glia contributes to ocular axial growth

**Fig 6:**
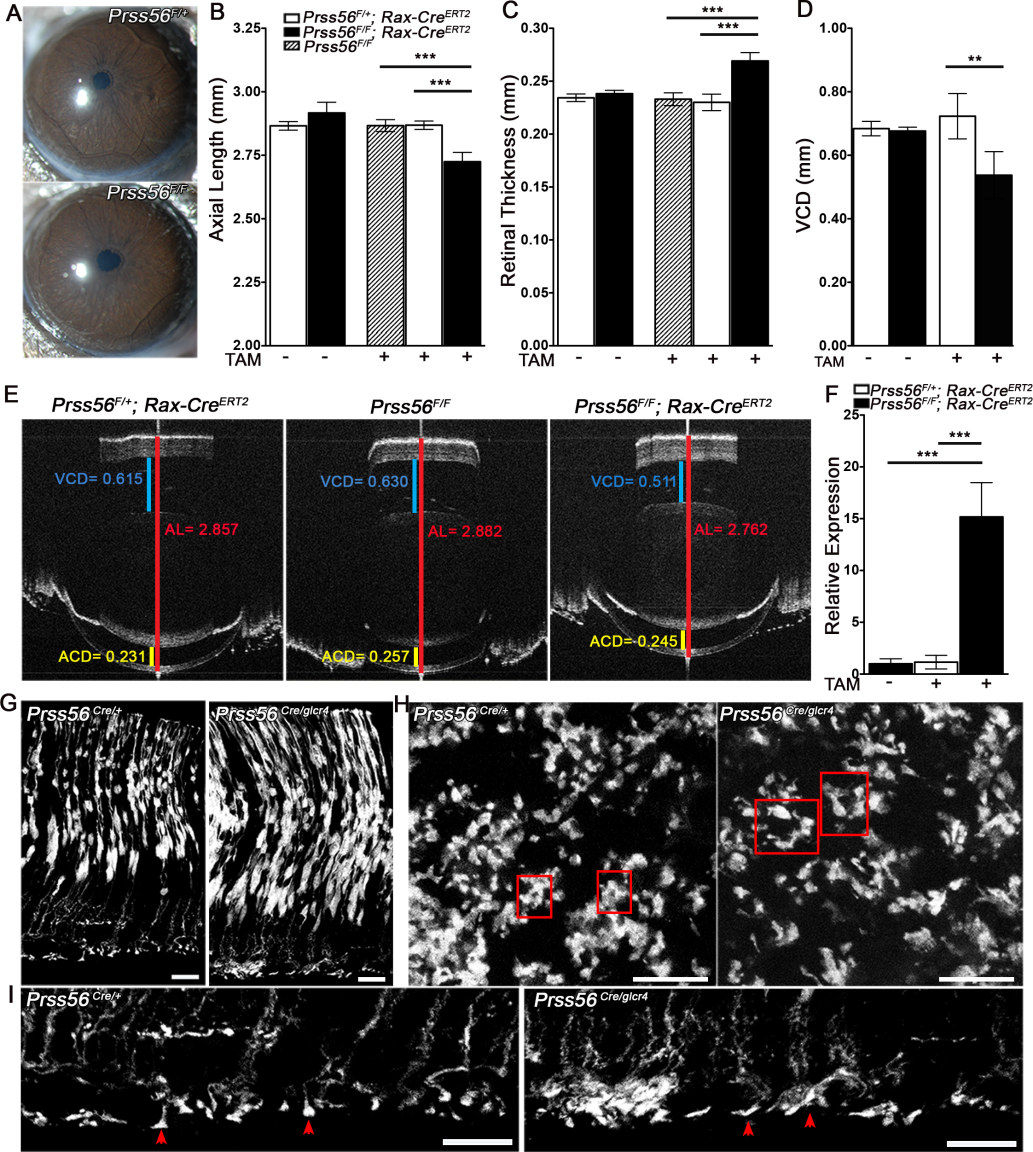
Conditional RAX-Cre-mediated ablation of *Prss56* from fully differentiated Müller glia leads to ocular size reduction. *Prss56* was conditionally ablated from Müller cells in a time-specific manner by crossing *Prss56*^*F/F*^ to the inducible RAX-Cre mouse strain (*Rax-Cre*^*ERT2*^). CRE expression was induced by tamoxifen injection at P8, a time point preceding complete Müller glia differentiation. (**A**) Representative images of slit lamp examination by broad-beam illumination following tamoxifen injection at P8. *Prss56*^*F/F*^*; Rax-Cre*^*ERT2*^ eyes were indistinguishable from control *Prss56*^*F/+*^*; Rax-Cre*^*ERT2*^ eyes at 2 months of age. (**B**, **C**) Following tamoxifen injection (TAM +; horizontal axis) at P8, *Prss56*^*F/F*^*Rax-Cre*^*ERT2*^ mice display a significant decrease in ocular axial length (**B**) and increase in retinal thickness (**C**) compared to control eyes (*Prss56*^*F/+*^*; Rax-Cre*^*ERT2*^ or *Prss56*^*F/F*^ mice without *Rax-Cre*^*ERT2*^), N = 6 to 8 per group. (**D**) Following tamoxifen injection at P8, a significant reduction in VCD was detected in *Prss56*^*F/F*^*; Rax-Cre*^*ERT2*^ eyes compared to control *Prss56*^*F/+*^*; Rax-Cre*^*ERT2*^ eyes, N = 6 per group. Ocular axial length, retinal thickness, and VCD in uninjected *Prss56*^*F/F*^*; Rax-Cre*^*ERT2*^ and *Prss56*^*F/+*^*; Rax-Cre*^*ERT2*^ mice were indistinguishable. (**E**) Representative OCT images showing reduced axial length and VCD in *Prss56*^*F/F*^*; Rax-Cre*^*ERT2*^ eyes compared to the control *Prss56*^*F/+*^*; Rax-Cre*^*ERT2*^ or *Prss56*^*F/F*^ mice. (**F**) qPCR analysis following tamoxifen injection at P8 revealed that *Prss56* mRNA was significantly upregulated in *Prss56*^*F/F*^ *; Rax-Cre*^*ERT2*^ retina compared to their *Prss56*^*F/+*^ *; Rax-Cre*^*ERT2*^ counterparts or uninjected controls, N≥ 6 per group. Values are presented as mean ±SD; * p<0.05, ** p<0.01, *** p<0.001, t-test. (**G-I**) Müller glia endfeet organization of *Prss56*^*Cre*^; *R26*^*tdTomato*^ reporter mice during retinal development. Representative images of retinal section (**G, I**) or whole mount (**H**) showing Müller glia endfeet from control and *Prss56* mutant mice at P6. (**I**) Magnified images of the retinal endfeet are shown. The ILM of *Prss56* mutant mice (*Prss56*^*Cre/gclr4*^; *R26*^*tdTomato*^) at P6 is marked by regions of increased endfeet complexity (arrow head) compared to the ILM of control mice (*Prss56*^*Cre/+*^; *R26*^*tdTomato*^). A substantial proportion of endfeet appear more spread out, occupying a larger area in the mutant compared to control retinal whole mounts (occupying smaller area). Red boxes highlight individual endfoot. N = 4 per genotype and scale bars = 17μm in **G** and **I**, and 50μm in **H**. ACD, anterior chamber depth; AL, axial length; VCD, vitreous chamber depth.

Ocular size reduction in *Prss56* mutant mice is detected as early as P6 when a significant pool of undifferentiated late RPCs is still present in the retina. Since *Prss56* is expressed by late RPCs, it raises the question of whether PRSS56 derived from either committed or differentiated Müller glia has any role to play in ocular axial growth. To address this, we generated a conditional *Prss56* mutant allele by flanking exons 2 to 4 with LoxP sites ***(****Prss56*^*F*^) that will give rise to a catalytically inactive PRSS56 protein following CRE-mediated excision (S4 Fig). Following validation of the conditional allele (S5 Fig), we ablated *Prss56* from Müller glia using the *Rax-Cre ER*^*T2*^ which specifically express CRE recombinase in Müller cells following tamoxifen induction [42]. Selective inactivation of *Prss56* in Müller glia was induced by tamoxifen injection at P8, a time point preceding the developmental stage at which the majority of RPCs have differentiated into their respective retinal cell types, including Müller cells (~ P10). Based on slit lamp examination, eyes from tamoxifen-injected *Prss56*^*F/F*^*; Rax-Cre ER*^*T2*^ and control *Prss56*^*F/+*^*; Rax-Cre ER*^*T2*^ mice were indistinguishable (Fig 6A). Ocular biometric analysis revealed a significant reduction in ocular axial length and VCD and an increase in retinal thickness in tamoxifen injected *Prss56*^*F/F*^*; Rax-Cre ER*^*T2*^ mice compared to both tamoxifen injected and uninjected controls (Fig 6B–6E). Similar to what we observed in the *Prss56*^*glcr4/glcr4*^ retina, we detected a significant upregulation of retinal *Prss56* mRNA levels following conditional ablation of *Prss56* from Müller glia compared to control eyes (Fig 6F). These findings suggest that PRSS56 derived from differentiated Müller cells contributes to ocular axial growth.

Next, we assessed if loss of PRSS56 function affects the structural or morphological organization of Müller glia. To this end, we took advantage of our *Prss56*^*Cre*^; *R26*^*tdTomato*^ reporter line, which specifically labels the subset of Müller glia derived from *Prss56*-expressing RPCs and performed a morphological analysis of retinal sections and whole-mounts at P6. This specific time point was selected for three main reasons: 1) it corresponds to the developmental time point when Müller glia committed progenitors begin to express markers of mature Müller glia [43], 2) it marks the initiation of endfeet formation and precedes complex endfeet elaboration in the inner limiting membrane (ILM) [44], and 3) it coincides with the time point at which ocular size reduction is first detected in *Prss56* mutant mice. Although we did not observe any gross change in the spatial arrangement of Müller glia in *Prss56* mutant retina (S3C Fig), we detected subtle alterations in the organization of Müller glia endfeet in P6 retinal sections and whole mounts (Fig 6G and 6H). We found that the proportion of tdTomato positive Müller glia endfeet exhibiting a more elaborate morphology was significantly greater in *Prss56* mutant compared to control retinal whole mounts (59.26 ± 8.04% in mutant vs 22.72 ± 5.33% in control, p<0.001). These findings suggest premature branching and maturation of Müller glia endfeet in *Prss56* mutant mice and raise the possibility that altered structural organization of Müller glia may contribute to the ocular size reduction observed in *Prss56* mutant mice (Fig 6G–6I).

#### Sustained PRSS56 activity is required for ocular axial growth

**Fig 7:**
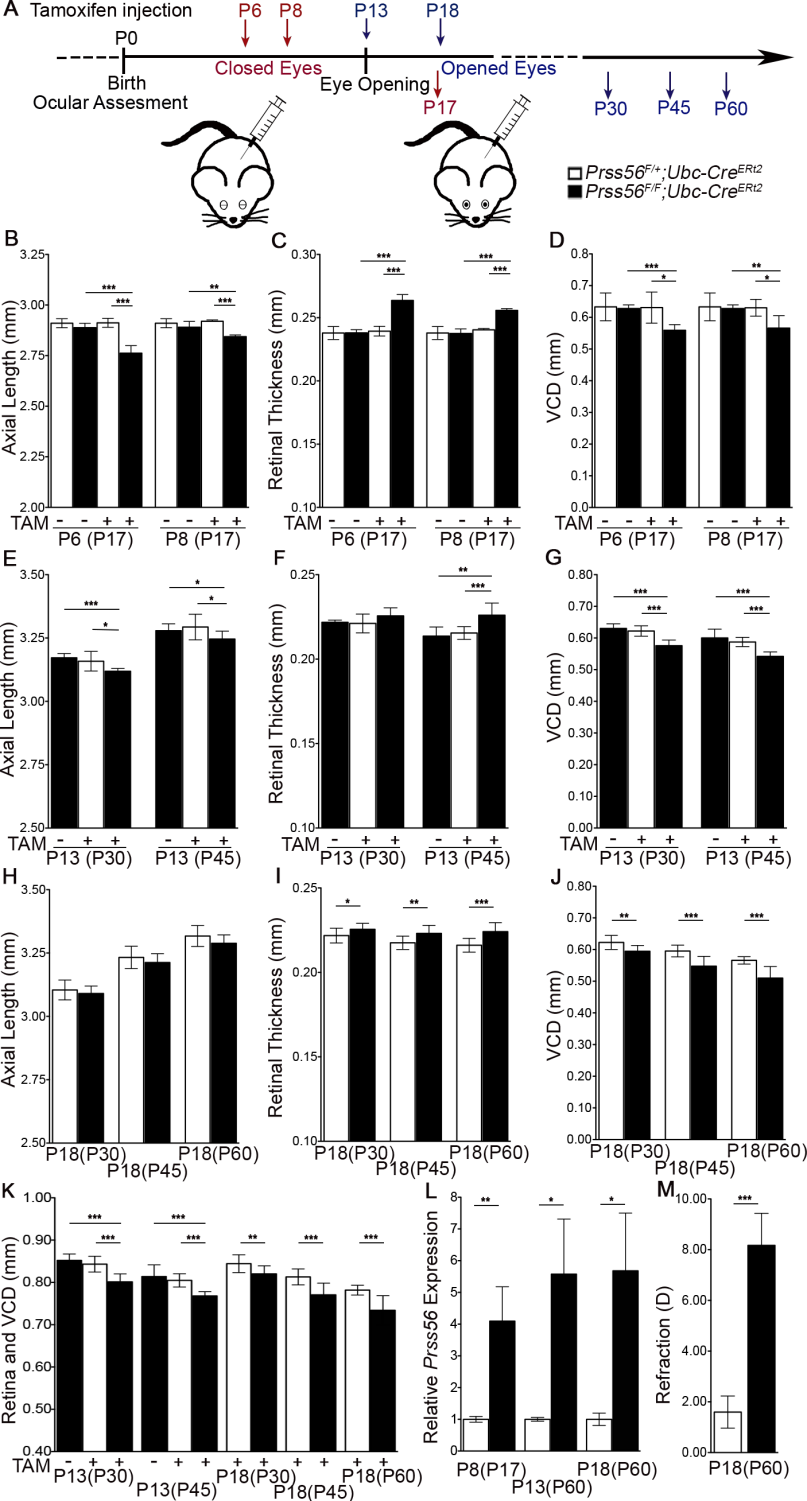
PRSS56 activity is required during both the vision-independent and dependent stages of ocular growth. To determine the temporal window critical for the PRSS56-mediated effect on ocular axial growth, *Prss56* conditional mutant mice (*Prss56*^*F/F*^) were crossed to mice expressing the ubiquitous inducible Ubc-Cre recombinase (*Ubc-Cre*^*ERT2*^). (**A**) Schematic of tamoxifen treatment at distinct developmental stages preceding and following the opening of the eyes. Tamoxifen injection at two different time points, P6 and P8, was performed to ablate *Prss56* after the earliest detectable effect of mutant *Prss56* on ocular axial length. (**B**-**D**) OCT-based ocular biometry demonstrates that following tamoxifen injection, *Prss56*^*F/F*^*; Ubc-Cre*^*ERT2*^ mice display a significantly reduced ocular axial length (**B**) and increased retinal thickness (**C**) and a significant decrease in VCD (**D**) compared to the control *Prss56*^*F/+*^; Ubc-Cre^*ERT2*^ mice (measured at P17). The ocular axial length, retinal thickness, and VCD of uninjected *Prss56*^*F/F*^*; Ubc-Cre*^*ERT2*^ *and Prss56*^*F/+*^*; Ubc-Cre*^*ERT2*^ mice were indistinguishable. Administration of tamoxifen at P6 caused a greater decrease in ocular axial length compared to administration at P8 suggesting a requirement for continuous PRSS56 activity during ocular development to sustain normal ocular growth, N = 6 to 8 per group for **A** and **B**. (**E**-**G**) OCT measurements demonstrate that following tamoxifen injection at P13 (a time point when the eyes are open), *Prss56*^*F/F*^*; Ubc-Cre*^*ERT2*^ mice display a slight but significant decrease in ocular axial length (**E**) and increase in retinal thickness (**F**) at P30 and P45. Reduced ocular axial length was associated with a significant decrease in VCD in *Prss56*^*F/F*^*; Ubc-Cre*^*ERT2*^ mice compared to *Prss56*^*F/+*^*; Ubc-Cre*^*ERT2*^ and uninjected controls at P30 and P45, N = 5 to 10 per group (**G**). **(H**-**J)** Ocular biometry following tamoxifen injection at the beginning of a critical emmetropization period (P18) shows that the ocular axial length is not significantly different between the *Prss56*^*F/F*^*; Ubc-Cre*^*ERT2*^ and control mice at any of the three ages examined (P30, P45, and P60). However, *Prss56*^*F/F*^*; Ubc-Cre*^*ERT2*^ mice display a slightly thicker retina and significantly reduced VCD compared to the control groups. (**K**) Following *Prss56* ablation at P13 and P18, the eyes display a decrease in the combined value of retinal thickness and VCD. (**L**) qPCR analysis revealed elevated *Prss56* mRNA levels in *Prss56*^*F/F*^*; Ubc-Cre*^*ERT2*^ retina compared to *Prss56*^*F/+*^*; Ubc-Cre*^*ERT2*^ retina following tamoxifen injection at P8, P13, or P18 (shown are data from mice harvested at P17 and P60, respectively). (**M**) Following *Prss56* ablation at P18, the eyes display a hyperopic shift in refraction compared to control eyes at 3 months (N = 6 per group). Values are presented as mean ± SD (or mean ± SEM in **L**); * p<0.05, ** p<0.01, *** p<0.001, t-test.

Our results show that *Prss56* is actively expressed in both the developing and adult retina, raising the possibility that PRSS56 might regulate ocular growth during distinct development stages spanning the pre and post-eye opening periods. To determine the temporal requirements of PRSS56 in modulating ocular axial growth, we bred conditional *Prss56* mutant mice to the inducible ubiquitous *Ubc-Cre* line to ablate *Prss56* at distinct stages of ocular development. Unlike the human eye, the mouse eye remains closed during the early postnatal period until the eyelids open around P13 (Fig 7A). Therefore, in this regard, postnatal time points preceding P13 in the mouse are analogous to late prenatal stages of human ocular growth when the eyes are closed. Mice carrying the *Prss56*^*F*^ allele and the inducible *Ubc-Cre* transgene (*Prss56*^*F/F*^; *Ubc-Cre ER*^*T2*^ and control: *Prss56*^*F/+*^*; Ubc-Cre ER*^*T2*^) were injected with tamoxifen at P6 or P8 (stages at which the eyes are closed) and compared to uninjected and injected control groups. Ablation of *Prss56* at both time points caused a significant decrease in ocular axial length and VCD compared to control groups (measured at P17; Fig 7B–7D). Administration of tamoxifen at P6 caused a greater reduction in ocular axial length (compare P6 to P8 in Fig 7B), suggesting a continuous requirement for PRSS56 activity during ocular growth. Next, we injected tamoxifen at P13 to determine whether PRSS56 function is required after eye opening. Ablation of *Prss56* following tamoxifen injection at P13 caused a modest but significant decrease in ocular axial length compared to control groups (measured at P30 and P45, Fig 7E). The decrease in ocular size was accompanied by a significant decrease in VCD and increase in retinal thickness (Fig 7F and 7G).

To test for a potential role of PRSS56 at stages when eyes are responsive to patterned visual stimulation [45], we injected *Prss56*^*F/F*^; *Ubc-Cre ER*^*T2*^ and control mice (*Prss56*^*F/+*^*; Ubc-Cre ER*^*T2*^) with tamoxifen at P18 and performed ocular biometry at 3 different time points (P30, P45, and P60). Although the axial length of *Prss56*^*F/F*^; *Ubc-Cre ER*^*T2*^ eyes tended to be slightly shorter compared to that of control eyes, the difference was not statistically significant at any of the ages examined (P = 0.056 at P60 measurement, Fig 7H). In contrast, a significant decrease in VCD was detected in *Prss56*^*F/F*^; *Ubc-Cre ER*^*T2*^ eyes compared to control eyes at all ages examined (Fig 7J). Notably, the decrease in VCD following conditional *Prss56* ablation was progressive and age-dependent. Since we did not observe a significant reduction in ocular axial length, we also measured other ocular layers contributing to ocular axial length, including the lens and anterior chamber. While the lens thickness was comparable between the *Prss56* ablated and control eyes (S6A Fig), the ACD was marginally increased following *Prss56* ablation (S6B Fig), adding to the difficulty of detecting small changes in ocular axial length. Additionally, we assessed ocular refraction at 3 months of age following tamoxifen injection at P18 and found that *Prss56*^*F/F*^; *Ubc-Cre ER*^*T2*^ mice are hyperopic compared to control mice (Fig 7M). Furthermore, a significant upregulation of retinal *Prss56* mRNA levels was also observed following tamoxifen injection at P13 and P18 (Fig 7L). A detailed summary of ocular biometric measurements, sample size, and statistical significance following conditional ablation of *Prss56* using *Rax-Cre* and *Ubc-Cre* is presented in S2 Table. Collectively, our findings indicate that sustained PRSS56 activity is required throughout distinct stages of ocular development to regulate axial growth. Significantly, they demonstrate that PRSS56 is a molecular factor that operates during both the pre- and post-eye opening developmental stages of ocular axial growth.

#### Genetic inactivation of PRSS56 rescues ocular axial elongation in a mouse model of myopia

**Fig 8:**
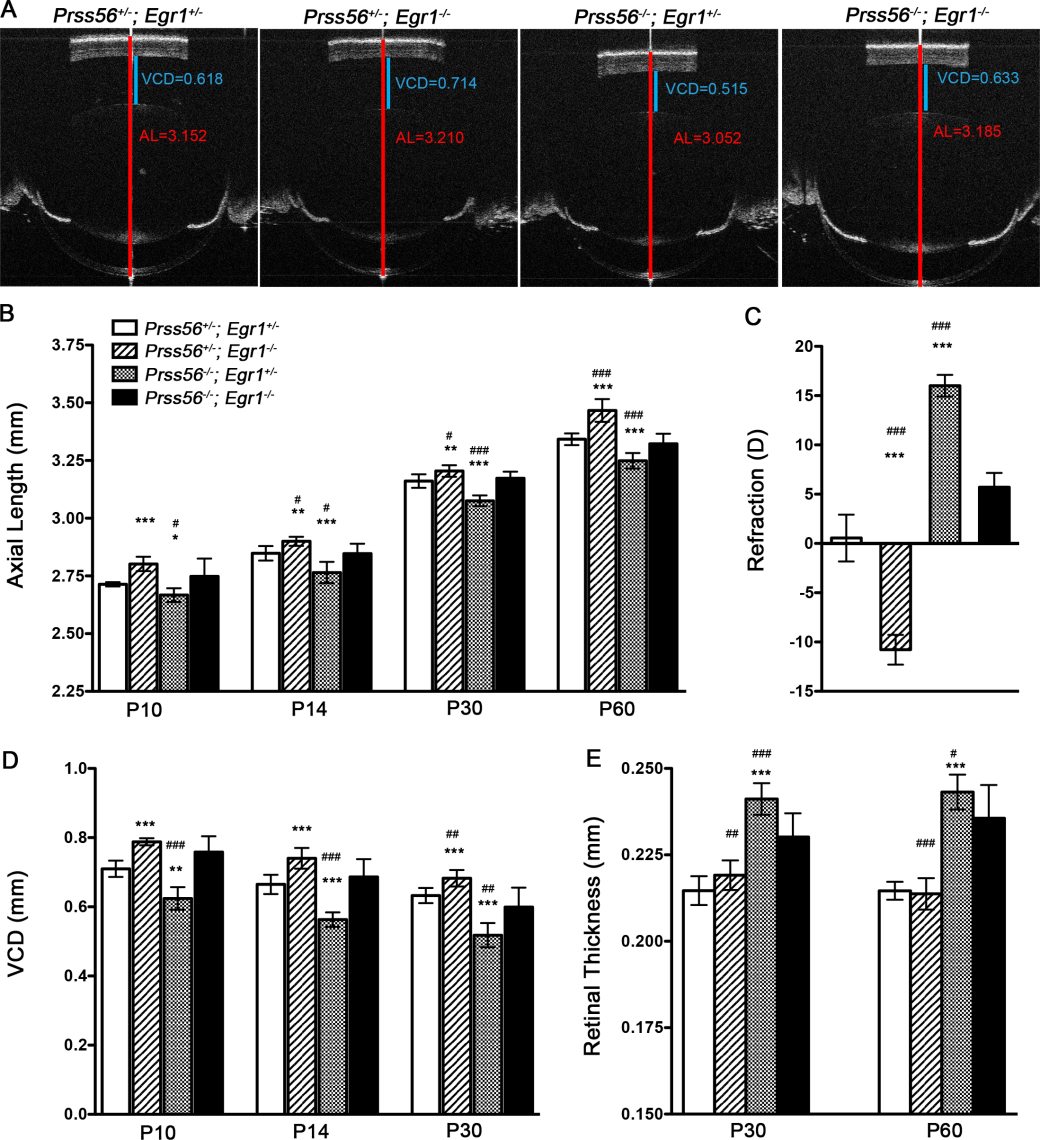
*Prss56* ablation rescues myopia in mice. (**A**) Representative OCT images demonstrating that *Prss56* ablation rescues myopia in *Egr1*^*-/-*^ mice (compare *Prss56*^*+/-*^*;Egr1*^*-/-*^ *to Prss56*^*-/-*^*;Egr1*^*-/-*^, shown are P30 eyes). Reciprocally, *Egr1* deficiency rescues hyperopia in *Prss56*^*-/-*^ mice (compare *Prss56*^*-/-*^*;Egr1*^*+/-*^ *to Prss56*^*-/-*^*;Egr1*^*-/-*^*)*. The red and blue lines indicate ocular axial length (AL) and vitreous chamber depth (VCD), respectively. (**B**) *Prss56*^*-/-*^*;Egr1*^*+/-*^ eyes display a significant reduction in axial length, whereas *Prss56*^*+/-*^*;Egr1*^*-/-*^ exhibit significantly elongated axial length compared to the control eyes (*Prss56*^*+/-*^*;Egr1*^*+/-*^ *)*. The eyes of double mutants (*Prss56*^*-/-*^*;Egr1*^*-/-*^*)* attain a size that is not significantly different from control eyes (*Prss56*^*+/-*^*;Egr*^*-/-*^), at all ages examined (P10 to P60). (**C**) Consistent with modulation of ocular axial length by *Prss56* and *Egr1* mutations, hyperopic refraction observed in *Prss56*^*-/-*^*;Egr1*^*+/-*^ eyes was rescued in the double mutants (*Prss56*^*-/-*^*;Egr1*^*-/-*^). Conversely, *Prss56* ablation rescued myopic refraction observed in *Prss56*^*+/-*^*;Egr1*^*-/-*^ eyes (compare *Prss56*^*+/-*^*;Egr1*^*-/-*^ *to Prss56*^*-/-*^*;Egr1*^*-/-*^, shown are data from 2-months old mice). (**D**) The VCD of double mutant eyes (*Prss56*^*-/-*^*;Egr1*^*-/-*^*)* was significantly reduced compared to *Egr1* single mutant eyes (*Prss56*^*+/-*^*;Egr1*^*-/-)*^ and increased compared to *Prss56* single mutant eyes (*Prss56*^*-/-*^*;Egr1*^*+/-*^). The VCD of double mutant eyes was not significantly different from the control eyes. (**E)** The retina is thicker in double mutants (*Prss56*^*-/-*^*;Egr1*^*-/-*^*)* compared to control mice *(Prss56*^*+/-*^*;Egr1*^*+/-*^) at P30 and P60, despite their ocular axial length being similar. Values are presented as mean ± SD. For comparison between single mutant and controls: * p<0.05; ** p<0.01;*** p<0.001, t-test. For comparison between single mutant and double mutants: ^#^ p<0.05; ^##^ p<0.01; ^###^p<0.001, t-test. **B**-**D**: N ≥ 6 per group; **E**: N ≥ 8 per group.

Our data shows that lack of PRSS56 activity cause ocular size reduction. To explore the translational potential of this finding, we tested the hypothesis that inactivation of PRSS56-mediated pathway(s) could slowdown axial elongation linked to myopia. To this end, we performed a genetic study to determine the effect of *Prss56* ablation in a mouse model of myopia caused by a null mutation in *Egr1*. EGR1 is a known regulator of ocular axial growth, and *Egr1* expression is up- or down-regulated in animal models with experimentally-induced reduction or increase in ocular axial length, respectively [46, 47] Importantly, mice lacking EGR1 (*Egr1*^*-/-*^) recapitulate the characteristic hallmarks of myopia including increased ocular axial length and a myopic refractive shift [31]. Because ocular biometric parameters were indistinguishable between *Prss56*^*+/-*^*; Egr1*^*+/-*^ and *Prss56*^*+/+*^*; Egr1*^*+/+*^ mice, we used *Prss56*^*+/-*^*; Egr1*^*+/-*^ mice as controls for our experiments (S7 Fig). The ocular axial elongation resulting from *Egr1* deficiency was detected as early as P10, but was more prominent at later time points (compare *Prss56*^*+/-*^*; Egr1*^*-/-*^ to *Prss56*^*+/-*^*; Egr1*^*+/—*^mice, Fig 8A and 8B). Importantly, a concomitant increase in VCD, but no significant change in retinal thickness was observed at all ages examined in *Egr1* deficient mice compared to control mice (Fig 8D and 8E). As expected, *Prss56* mutant eyes (*Prss56*^*-/-*^*; Egr1*^*+/-*^) exhibit a significant reduction in axial length and VCD compared to control eyes (*Prss56*^*+/-*^*; Egr1*^*+/-*^, Fig 8B–8D). Interestingly, the axial length and VCD of double mutant (*Prss56*^*-/-*^*; Egr1*^*-/-*^*)* eyes were not significantly different from that of the control eyes, (Fig 8A, 8B and 8D). Consistent with our ocular size findings, refraction was significantly rescued in double mutants compared to single *Egr1* or *Prss56* mutants, exhibiting myopia or hyperopia, respectively (Fig 8C). Interestingly, despite the ocular axial length of double mutants being comparable to that of control mice, they exhibit a significantly thicker retina, similar to what is observed in *Prss56* single mutant eyes (Fig 8E). A detailed summary of ocular biometric measurements from *Egr1* and *Prss56* single and double mutants, sample size, and their statistical significance are presented in S3 Table. These findings show that *Prss56* inactivation rescues axial elongation/myopia resulting from EGR1 deficiency.

### Discussion

The molecular and cellular mechanisms involved in ocular size regulation and refractive development are poorly understood. Here, we have used a combination of genetic mouse models to elucidate the role of PRSS56 in ocular axial growth. We show that loss of PRSS56 function causes ocular axial length reduction and hyperopia. Moreover, utilizing a lineage tracing strategy and a combination of molecular approaches, we demonstrate that *Prss56* ocular expression is first detected in a pool of late RPCs and then in a subset of Müller glia following retinal cell differentiation. Importantly, our findings demonstrate that PRSS56 derived from Müller glia contributes to ocular axial length elongation, uncovering a previously unrecognized role for Müller glia in ocular growth. Furthermore, we show that continuous PRSS56 activity is required to sustain ocular growth throughout distinct stages of ocular development spanning the pre- and post-eye opening periods. Thus, findings from our mouse models suggest that at least some of the factors guiding ocular growth are conserved across the prenatal and postnatal stages of ocular development.

#### Impaired processing of PRSS56’s endogenous substrate(s) potentially contributes to ocular size reduction

Using mice homozygous for a null allele of *Prss56*, we demonstrate that loss of PRSS56 function causes a reduction in ocular size and hyperopia (Fig 1A–1F), suggesting that impaired processing of PRSS56 protease’s endogenous substrate(s) may underlie ocular size reduction. Since the loss of PRSS56 function leads to reduced ocular size, *PRSS56* variants associated with myopia (identified by GWAS) likely act in an opposite manner, i.e. via a gain of function mechanism, to induce ocular axial elongation [16, 40, 48]. Our current efforts are geared towards identifying PRSS56 endogenous substrates to gain further mechanistic insight into factors contributing to ocular growth.

#### Identification of a novel subpopulation of Müller glia

Our results demonstrate that *Prss56* ocular expression is first detected embryonically in a pool of late RPCs and later in Müller glia following retinal cell differentiation (Figs 2 and 3). Müller glia has traditionally been viewed as a homogeneous cell population. Although previous studies have reported heterogeneity in gene expression among individual Müller cells, evidence supporting Müller glia being a heterogeneous group of cells is very limited [49, 50]. Here, we have identified a unique subset of Müller glia that expresses *Prss56* and are enriched in the peripheral region of the retina (Figs 2, S3B and S3C). It is tempting to speculate that the heterogeneity in the molecular profile and distribution of Müller cells may have evolved to support their diverse retinal functions.

#### Modulation of *Prss56* expression in response to mutations in gene(s) involved in ocular size determination

Our study demonstrates that retinal expression of *Prss56* is significantly increased in response to loss of PRSS56 function (observed both in *Prss56*^*glcr4/glcr4*^ mice or following conditional *Prss56* ablation, Figs 4A and 4B, 6F and 7L). Interestingly, retinal expression of *Prss56* is also upregulated in *Mfrp* mutant mice [51]. Mutations in the gene coding for MFRP, a frizzled-related protein expressed in the retinal pigment epithelium (RPE), cause nanophthalmos in humans, a phenotype similar to that resulting from *PRSS56* mutations [13]. These findings suggest that modulation of *Prss56* expression levels may have evolved as part of a feedback regulatory mechanism aimed at overcoming alterations in ocular growth resulting from disruption of genes/pathways involved in refractive development.

#### Identification of a novel role of Müller glia in ocular axial growth

In this study, we used conditional *Prss56* mutant mice to demonstrate that PRSS56 derived from differentiated Müller glia contributes to ocular axial growth (Fig 6A–6F). However, ocular size reduction in *Prss56* mutant mice is detected before complete differentiation of Müller glia, suggesting that both glial committed progenitor cells and differentiated Müller cells constitute important sources of PRSS56 to support ocular growth. Being a secreted protease, PRSS56 could modulate the extracellular milieu of the retina by processing its endogenous substrate(s), such as retinal or neighboring ECM components or cell surface receptors. Notably, PRSS56 was recently shown to be localized in the retinal ILM (the retinal basement membrane) [52]. Thus, it is possible that loss of PRSS56 activity could alter the composition of the ILM, which in turn could lead to the premature elaboration of Müller glia endfeet observed in *Prss56* mutant retina (Fig 6G–6I). Defective ILM has been linked to abnormal ocular growth and mutations in genes encoding ECM proteins present in the ILM have been implicated in axial elongation and myopia [53]. Henceforth, the ILM has been proposed as a critical structure providing the mechanical strength regulating the pressure within the vitreous chamber, which is subsequently transferred to the sclera of the developing eye to modulate ocular growth [53]. It is tempting to speculate that failure to generate sufficient tension due to altered ILM composition may underlie ocular size reduction resulting from loss of PRSS56 function. Alternatively, as reduced VCD was the most conspicuous ocular manifestation detected in mice and a patient with mutant PRSS56 (Fig 1E and 1I), a failure to maintain normal vitreous volume may account for the altered ocular axial length. Müller glial cells have been suggested to be important regulators of intraretinal water flow into the vitreous cavity [54–56]. Thus, another possible mechanism by which loss of PRSS56 function may contribute to ocular size reduction is by failure to regulate fluid flow and maintain normal vitreous volume.

#### Findings from our mouse study suggest a role for PRSS56 in pre- and post-natal human ocular growth

Our results suggest that continuous PRSS56 activity is required for normal ocular growth. Developmental stages preceding and following eye opening in the mouse are analogous to the prenatal and postnatal stages (when the eyes are exposed to visual experience) of human ocular growth, respectively. Thus, the impact of *PRSS56* mutations on human ocular growth is likely to begin prenatally before patterned visual activity modulates the rate of ocular growth. These findings are in agreement with previous suggestions that genetic alterations leading to nanophthalmos interfere with prenatal ocular growth [14]. Importantly, ablation of *Prss56* following eye opening in the mouse (tamoxifen injection at P13 and P18) resulted in a modest reduction in ocular dimension and a hyperopic shift in refraction (Fig 7E, 7J and 7M), suggesting that the role of PRSS56 in ocular growth regulation may extend beyond the prenatal developmental window in humans. These findings establish that persistent PRSS56 activity is required during distinct stages of ocular development to support ocular growth and normal refractive development. Although, visual input plays a critical role in the regulation of postnatal ocular growth, both vision-adjusted and unadjusted ocular growth are likely to operate in concert to determine ocular size [17, 57]. Here, we have discovered that PRSS56 contributes to ocular growth during a window when the eyes are subject to patterned visual stimulation (Fig 7E–7J). The specific contribution of PRSS56 in vision-adjusted ocular growth, if any, is yet to be determined. However, it is plausible that the fundamental processes controlling ocular growth are conserved throughout the various stages of ocular development [17], with an additional level of regulation during the vision-adjusted phase where the rate of ocular growth is dependent on the refractive status of the eye. Future studies aimed at investigating the specific role of PRSS56 in emmetropization will utilize experimental paradigms to examine the effect of stage-specific ablation of *Prss56* on vision-guided ocular growth.

#### Identification of PRSS56 as a potential target for therapeutic intervention to slowdown myopia progression

The most common forms of myopia result from aberrant postnatal ocular growth. Given the role of PRSS56 in supporting ocular growth at stages following opening of the eyelid, targeting PRSS56 appears a viable therapeutic strategy to slowdown axial elongation underlying myopia. Our genetic experiment suggests that opposing effects of *Prss56* and *Egr1* mutations are canceled in double mutant animals, causing their eyes to attain a size that is indistinguishable from that of control eyes. Thus, it is likely that parallel reciprocal pathways driven by PRSS56 and EGR1 regulate ocular axial growth. Interestingly, EGR1 is detected in cells of the retinal INL, including amacrine cells [47], raising the possibility that PRSS56 and EGR1 may act through two different retinal cell types to regulate ocular size. Overall, these results demonstrate that *Prss56* inactivation rescues axial elongation/myopia resulting from EGR1 deficiency, and thereby establish PRSS56 as a potential therapeutic target for interventions aimed at preventing myopia.

In summary, we demonstrate that loss of PRSS56 function leads to ocular size reduction and hyperopia and identify a novel role for Müller glia in ocular axial growth. Future studies will aim at determining whether PRSS56 regulate ocular growth by directly influencing Müller glia function. Our findings also show that PRSS56 activity is required at distinct developmental stages spanning the pre- and post-eye opening periods. To the best of our knowledge, this is the first study documenting the existence of a genetic factor whose activity is required continuously through various stages of ocular development to support normal refractive development. Finally, we demonstrate that *Prss56* inactivation rescues axial elongation in a mouse model of myopia. Since *PRSS56* variants have been implicated in both human hyperopia and myopia, our findings have direct relevance to human ocular refractive development. Given the steep rise in the prevalence of myopia, there is an urgent public health need to identify therapeutic targets to prevent or slow down myopia. This study demonstrates that interventions aimed at regulating PRSS56 activity have the potential to modulate ocular growth, restore healthy refractive development and prevent associated blinding conditions.

## Methods

### Ethics statement

All experiments were conducted in accordance with the Association for Research in Vision and Ophthalmology’s statement on the use of animals in ophthalmic research. Mouse studies were performed in compliance with protocols approved by the Institutional Animal Care and Use Committee at University of California San Francisco (Approval numbers: AN153083 and AN120008). Animals were given access to food and water ad libitum and housed under controlled conditions including a 12-h light/dark cycle in accordance with the National Institutes of Health guidelines. For some of the experiments mice were anesthetized with ketamine/xylazine (100 mg/kg and 5mg/kg, respectively).

### Mouse lines

#### Mutant mice

*Prss56*^*glcr4*^: C57BL/6.Cg*-Prss56* ^*glcr4*^*/SjJ*: Mice carrying ENU induced mutation in *Prss56*, causing truncation of PRSS56 C–terminal region [36].

*Prss56* targeted mutation (*Prss56*^*Cre*^): C57BL/6.Cg-*Prss56*^*tm(cre)*^, *Prss56* exon1 was replaced by a sequence coding for CRE recombinase to generate a null mutation [41]. *Prss56* conditional mutation (*Prss56*^*F*^): C57BL/6.Cg-*Prss56*^*tm1*^*/*SjJ, mice carrying LoxP sites flanking exons 2 to 4 of *Prss56*. Excision of the LoxP sites results in a catalytically inactive form of PRSS56.

*Egr1* mutant mice: C57BL/6.*Egr1*^*tm1Jmi*^/J, the targeted mutation by insertion of a PGK-neo cassette introduces stop codon resulting in protein truncation upstream of the DNA-binding domain [58].

#### Reporter line

*Gt(ROSA)26Sor*^*tm14(CAG-tdTomato)Hze*^: *Cre* reporter mice harbor a LoxP-flanked STOP cassette preventing transcription of a CAG promoter-driven tdTomato [59]. The targeted mutation was inserted into the Gt(ROSA)26Sor locus. TdTomato expression is dependent on the presence of CRE recombinase.

#### Cre lines

1. *Rax-Cre ER*^*T2*^: *Rax*^*tm1*.*1(cre/ERT2)Sbls*^/J mice express tamoxifen-inducible CRE recombinase under the control of the *Rax* promoter [42].
2. *Ubc-Cre ER*^*T2*^: C57BL/6.Cg-*Tg*(*UBC-Cre/ER*^*T2*^)*1Ejb*, Cre-ERT2 transgenic mouse line was generated carrying a human ubiquitin C (UBC) promoter sequence upstream of a Cre-ER^T2^ fusion gene [60].
3. *Sox2-Cre*:*Tg(Sox2-cre)1Amc/J*: Sox2-Cre transgenic mice express CRE recombinase under the control of the mouse *Sox2* promoter expressed as early as E6.5 (ubiquitous Cre line) [61].

No gender effect has been observed on ocular size reduction resulting from *Prss56* mutations in our previous and current studies. Both males and females were included in all experiments described in this manuscript. For most experiments, littermates were used for comparisons between genotypes. PCR genotyping was performed on genomic DNA obtained from tail biopsies digested with Proteinase K (Sigma, St. Louis, MO, USA) using the primers indicated in S4 Table.

### Clinical slit lamp examination

Ocular anterior segment examinations were performed on 1–5 months old mutant mice and control littermates using a slit lamp biomicroscope (Topcon SL-D7; Topcon Medical Systems, Oakland, NJ, USA) attached to a digital SLR camera (Nikon D200; Nikon, Melville, NY, USA). Observers were masked to mouse genotypes while evaluating clinical phenotypes. Phenotypic evaluation included considerations for iris structure, pupillary abnormalities, cataracts and the overall dimensions of the anterior chamber.

### Ocular biometry

Ocular biometry was performed using optical coherence tomography or a digital Vernier caliper. Envisu R4300 spectral-domain optical coherence tomography (SD-OCT, Leica/Bioptigen Inc., Research Triangle Park, NC, USA) was employed to measure the ocular axial length, retinal thickness, vitreous chamber depth (VCD) and anterior chamber depth (ACD) as previously described with minor modifications [25]. Briefly, mice were anesthetized with ketamine/xylazine (100 mg/kg and 5mg/kg, respectively; intraperitoneal) and their eyes dilated before placing the animal in a cylindrical holder. The eye was hydrated with Genteal (Alcon, Fort Worth, TX, USA) and positioned in front of the OCT light source. Correct alignment of the eye was achieved by placing the Purkinje image in the center of the pupil. The images were acquired in rectangular volume and radial volume scans to capture the retinal thickness and axial length measurements, respectively. The axial length was calculated by measuring the distance from the corneal surface to the RPE/choroid interface. The distance between the innermost layer of the retina and the lens was used to calculate the VCD. ACD is the distance between the innermost cornea layer and lens. For biometric analyses performed before eye opening (before P13), mice were anesthetized and their eyelid carefully slit open using fine scissors. Thereafter, the eye was gently protruded using a Q-tip and aligned to the light source as described above. Digital Vernier caliper (Fowler Ultra-Cal Mark III) was used to measure the equatorial diameter as described previously [36]. Eyes were enucleated and magnified under a dissecting scope. The Vernier caliper was positioned along the nasal and temporal plane at a point of maximum diameter. Ocular biometry was performed on both the left and right eyes of a given mouse. S1–S3 Tables summarize the details about sample size, body weight, and biometric measurements of mice in each experimental cohort. To minimize the possible effect of body weight on ocular size, we ensured that body weight of littermates was within a narrow range in each of the comparative groups.

### Refraction measurement

Ocular refractions were acquired using an automated infrared photorefractor as described previously with some minor modifications [28]. Refraction was measured following treatment of mouse eyes with cyclopentolate (Alcon, Fort Worth, Tx, USA) to temporarily paralyze the ciliary body (cycloplegic refraction). Mice were placed on a pedestal with their eyes facing the photorefractor. The photorefractor was maintained at a distance and maneuvered to obtain a clear focused image of the eye. The photorefractor registers a successful refraction measurement only when the Purkinje image is positioned in the center of the pupil as detected and marked by a green LED flash. Centering of the Purkinje image ensures the infrared rays pass along the optical axis. In a typical recording, around 30–50 refraction measurements are acquired, which are then used to calculate the mean OD. Refraction measurements were performed on both the left and right eyes of a given mouse. A total of at least 6 eyes per experimental group were used for refraction measurements and experimental mice of both sexes were used for refraction measurements.

### Quantitative real-time polymerase chain reaction (qRT-PCR)

Eyes were enucleated and retinas were immediately dissected. Total RNA was isolated from retina using Qiagen RNeasy Mini Kit with on-column DNase I treatment (Qiagen, Valencia, CA, USA) and reverse transcribed using iScript cDNA Synthesis Kit (Bio-Rad, Hercules, CA, USA*)*. qPCR was performed on a Bio-Rad C1000 Thermal Cycler/CF96 Real-Time System using SsoAdvanced^TM^ SYBR Green^®^ Supermix (Bio-Rad, Hercules, CA, USA), and primer sets listed in S5 Table. Briefly, 15ng of cDNA and 0.25 μM primers were used per reaction in a final volume of 10 μl of Supermix. Each cycle consisted of denaturation at 95°C for 5s, followed by annealing and extension at 60°C for 25s. Each reaction was run as technical duplicates and a minimum of 4 biological replicates was used per group. The relative expression level of each gene was normalized to housekeeping genes (*Actb*, *Hprt1*, and/or *Mapk1*) and analyzed using the CFX manager software (Bio-Rad, Hercules, CA, USA).

### Lineage tracing and Müller glia endfeet assessment using the Prss56^Cre^; R26^tdTomato^ reporter mice

*Prss56*^*Cre*^ mice were bred to tdTomato reporter mice (*R26*^*tdTomato*^) to generate offsprings with one copy of each of tdTomato and Cre recombinase under the control of the *Prss56* promoter. The offsprings (heterozygous control *Prss56*^*Cre/+*^; *R26*^*tdTomato*^) were utilized for *Prss56* lineage tracing and expression analysis. The eyes were enucleated at both embryonic and postnatal time points. The eyes were processed, sectioned and visualized for tdTomato fluorescence as described below. To assess tdTomato expression (S3 Fig) and organization of Müller glial endfeet (Fig 7G and 7H) in a *Prss56* mutant context (homozygous mutant), we designed our breeding strategy such that *Prss56* mutant mice carried a single copy of both tdTomato and *Prss56*^*Cre*^ (similar to control mice) and a copy of the *Prss56*^*glcr4*^ allele (*Prss56*^*Cre/glcr4*^; *R26*^*tdTomato*^). Our design ensured uniform copy number of tdTomato and *Prss56*^*Cre*^, allowing a direct comparison of tdTomato expressing cells between *Prss56* mutant and heterozygous control eyes.

### Immunofluorescence

Eyes were enucleated and immersion-fixed in 4% paraformaldehyde (PFA) in phosphate buffered saline (PBS) overnight at 4°C, cryoprotected in 20% sucrose in PBS, and embedded in Optimal Cutting Temperature (O.C.T.) compound (Tissue-Tek; Sakura Finetek, Torrance, CA, USA). Twelve micron cryosections were immunolabeled with anti-Sox2 (1:500 dilution, goat, cat#AF2018, R&D systems, MN, USA), or anti-PKCα (1:250, rabbit, P4334, Sigma, St. Louis, MO, USA) or anti-Vimentin (1:100 dilution, mouse IgM, clone 40E-C, IA, DSHB, USA), antibodies in PBS containing 10% normal donkey serum, 0.1% TritonX-100 (PBS-T). Immunolabeling was visualized using AlexaFluor 594 or 488 conjugated secondary antibodies raised in donkey (1:500, Life Technologies, Carlsbad, CA, USA) in PBS-T. Slides were mounted in Mowiol containing DAPI (2 μg/ml).

### *In situ* hybridization

Mice were transcardially perfused with ice-cold RNase-free PBS followed by 4% PFA (in RNase-free PBS). Enucleated eyes were post-fixed in RNAse-free 4% PFA, cryoprotected in 20% sucrose, and embedded in OCT and sectioned within 24 hours for in situ hybridization. QuantiGene View RNA (Affymetrix, Santa Clara, CA, USA) in situ hybridization assay was performed according to the manufacturer protocol. Briefly, 12μm cryosections were fixed overnight in 4% PFA, dehydrated through a graded series of ethanol, were subjected to 2X protease digestion for 10 minutes, postfixed with 4% PFA and hybridized with probe sets against the gene of interest for 3 hours at 40°C using a ThermoBrite system (Abbott Molecular, Des Plaines, IL, USA). Cryosections were then washed and subject to signal amplification and detection using fast red substrate, counterstained and mounted for subsequent imaging. For dual fluorescent *in situ* hybridization, Digoxigenin- and Fluorescein-labeled riboprobes were synthesized from full-length cDNA clones (MGC Mouse glutamine synthetase cDNA Clone Id:4224865). The hybridized mRNA was detected using the TSA-FITC/ TSA-CY5 Tyramide Signal Amplification System (PerkinElmer, Waltham, MA, USA).

### Tamoxifen injection

Tamoxifen (T-5648, Sigma, St. Louis, MO, USA) was dissolved in ethanol (200mg/ml) and diluted in corn oil (final concentration of 20mg/ml tamoxifen). Each experimental mouse received a single intraperitoneal injection of tamoxifen (0.6 mg or 30 μl of tamoxifen solution/mouse for P6 and P8 pups and 0.8 mg or 40 μl for P13 and P18 mice).

### Histology

Mice were euthanized and eyes enucleated and immediately immersed in cold fixative (1% PFA, 2% glutaraldehyde, and 0.1 M cacodylate buffer) for 24 hours, after which they were transferred to cold 0.1 M cacodylate buffer solution for an additional 24 hours. Samples were embedded in glycol methacrylate, and serial sagittal sections (2μm) passing through the optic nerve were cut and stained with hematoxylin and eosin (H&E).

### Retinal whole mount preparation and endfeet assessment

Retina was dissected from *Prss56*^*Cre/+*^; *R26*^*tdTomato*^ (control) or *Prss56*^*Cre/glcr4*^; *R26*^*tdTomato*^ (mutant) mice and four radial incisions made and mounted on a slide. tdTomato positive terminal endings of Müller glia projection (endfeet) were visualized and images captured using a Confocal mircroscope (Carl Zeiss LSM700). Eight equivalent areas of the retina were consistently selected for each whole mount. Müller glia endfeet were classified into two groups based on their morphology: 1) Müller glia endfeet showing simple cohesive arrangement and occupying smaller area of the retina. 2) Müller glia endfeet exhibiting more spread-out morphology and covering a larger area, suggestive of increased branching and elaboration. Two independent observers masked to genotypes manually quantified the relative distribution of the two types of endfeet.

### Retinal cell suspension

Eyes were enucleated and retina was isolated and minced in DMEM (Dubelcco’s Modified Eagles Medium, Gibco-Invitrogen Corporation, Carlsbad, CA, USA). Retina was then dissociated in 15IU papain (Worthington Biochemicals Freehold, NJ, USA) and 20μg/ml DNase I (Roche Applied Science, Mannheim, Germany) for 30 minutes at 37°C, gently triturated using a glass Pasteur pipet and passed through a 40 μm cell strainer. Tissue trapped by the strainer was digested with 1 mg/ml collagenase type I (Worthington Biochemicals Freehold, NJ, USA) and 15 μg/ml DNAse I (Roche Applied Science, Mannheim, Germany) for 30 min at 37°C. Flow-through was mixed with DMEM with 10% fetal bovine serum (FBS, Gibco*-*Invitrogen Corporation, Carlsbad, CA, USA) and washed 2X (300g for 2 minutes at RT). The retinal cell suspension was used for flow cytometry.

### Flow cytometry

Retinal cell suspension was fixed in 4% PFA and subjected to indirect immunolabeling using anti-GS (mouse, 1:500, MAB302, EMD Millipore, Billerica, MA, USA) or anti-Rhodopsin (mouse, 1:1000, MAB5336, EMD Millipore, Billerica, MA, USA) and fluorochrome labeled secondary antibodies (AlexaFluor 488 conjugated secondary antibodies raised in donkey, 1:500, Life Technologies, Carlsbad, CA, USA) in 10% NDS/PBS containing 0.1% Triton X-100 at 4°C. Flow cytometry of immunolabeled cell suspension was performed using a BD™ LSRII Fortessa flow cytometer and FACS Diva Software (BD Biosciences, San Jose, CA). Retinal cell suspension from *Prss56*^*Cre/+*^; *R26*^*TdTomato/+*^ mice incubated with AlexaFluor 488-conjugated secondary antibody were used as negative controls to establish gating parameters.

### Microscopy

Bright-field images were captured using AxioVision software and an AxioImager M1 microscope equipped with an AxioCam ICc3 digital camera (Carl ZeissMicroscopy, LLC, Germany). Fluorescent images were acquired using AxioImager M1 microscope equipped with an MRm digital camera and AxioVision software, with an LSM700 confocal microscope and a Zen software (Carl Zeiss Microscopy, LLC, Germany). Amira software was used for 3D visualization and analysis.

### Human ocular biometry

We obtained illustrative biometric data from a nanophthalmic patient affected with a homozygous missense variant (p.G320R) in *PRSS56* previously identified by one of the authors (ACO) [38], and also a representative normal volunteer for comparison. Approval for this study was obtained from the Research Ethics Board of the Nova Scotia Health Authority, Halifax, Nova Scotia, Canada. In brief, ocular dimensions measured via ultrasound biomicroscopy A- and B-scan techniques revealed a very small globe bilaterally featuring crystalline lenses that were normally positioned, but large in size relative to that of the eye. The choroid was also observed to be diffusely thickened.

### Statistical analysis

Statistical comparisons between control and mutant samples were performed by a two-tailed unpaired Student’s t-test using Prism version 6.0f software. p values of <0.05 were considered significant. Power for a two-tailed two-sample t-tests was calculated using a range of means and standard deviation values of axial length that could be reasonably expected based on published data and our initial assessment [26]. Although the difference between the two group means and within-group standard deviation are statistically independent parameters, in many biological data sets they show various levels of collinearity. Therefore, for small, medium and large μ1- μ 2 values, we based our power calculation on correspondingly increasing expectations of SD. The detection of differences with 80% power is possible in these scenarios with sample sizes ≤ 7. For example, with respect to axial length, a mean difference of 50 μm, SD = ± 30, the effect size is 1.67 requiring a sample size of 6. Where logistically possible, and partly as a precaution against the possibility of some failed experiments, we collected data from more samples. Number of eyes, mean and standard deviation of all measurements in each group are presented in S1–S3 Tables.

## Supporting information

S1 Fig Ocular dimensions of *Prss56*^*+/-*^ and *Prss56*^*+/+*^ mice are indistinguishable and significantly different from those of *Prss56*^*-/-*^ mice. (**A**, **B**) Histograms showing that ocular axial length (**A**) and retinal thickness (**B**) are indistinguishable in *Prss56*^*+/-*^ and *Prss56*^*+/+*^ mice at 3 months of age. (**C**) Representative images of enucleated eyes showing a modestly reduced size in *Prss56*^*-/-*^ compared to *Prss56*^*+/-*^ mice (shown are P15 eyes). (**D**) Representative optical coherence tomography images of 2 months old eyes. (**E**-**H**) Histograms showing reduced ocular axial length (**E**), increased retinal thickness (**F**), increased anterior chamber depth (ACD), decreased vitreous chamber depth (VCD) (**G**) in *Prss56*^*-/-*^ eyes compared to *Prss56*^*+/-*^ mice at 2 months of age. (**H**) Lens thickness was indistinguishable between *Prss56*^*-/-*^ and *Prss56*^*+/-*^ eyes. Data are presented as mean ± SD, ***p<0.001, t-test. In (**A**) N = 6 and 4 for *Prss56*^+/-^ and *Prss56*^+/+^, respectively; in (**E**-**H**) N = 10 and 9 for *Prss56*^*+/-*^ and *Prss56*^-/-^, respectively.
(TIF)

S2 Fig *Prss56* ocular expression is restricted to the retina. (**A**-**D**) Representative images of *Prss56*^*Cre/+*^;*R26*^*tdTomato/+*^ and *Prss56*^*+/+*^;*R26*^*tdTomato/+*^ ocular sections showing that tdTomato labeling (in red, reporting *Prss56* expression) is restricted to the retina in *Prss56*^*Cre/+*^;*R26*^*tdTomato/+*^ mice. (**B**) tdTomato was not detected in the absence of Cre expression (*Prss56*^*+/+*^;*R26*^*tdTomato/+*^). tdTomato-labeled cells were enriched in the peripheral region and relatively sparser in the central region of the retina (**A**, **C**, the peripheral and central regions of the retina are oriented left to right). (**C**, **D**) tdTomato expression was not detected in the iridocorneal angle, ciliary body (CB), cornea, lens, sclera, choroid or retinal pigment epithelium (RPE). Scale bars = 100μm.
(TIF)

S3 Fig *Prss56* expression in *Prss56* mutant and control retina. (**A**) Representative image of P20 *Prss56*^*Cre/+*^;*R26*^*tdTomato/+*^ retinal sections immunolabeled for the glial cell marker vimentin, showing colocalization of tdTomato and vimentin (arrows). (**B**) Detection of *Prss56* mRNA expression at P10 and P15 using QuantiGene View RNA *in situ* hybridization. Top panel: A representative image of P10 retina showing *Prss56* expression predominantly in the peripheral region of the retina. At P15, increased *Prss56* expression was detected in the inner nuclear layer of the retina in mutant mice compared to their wild-type littermates. (**C**) Representative P30 eye sections showing an increased number of tdTomato positive cells in a mutant *Prss56*^*Cre/glcr4*^;*R26*^*tdTomato/+*^ retina compared to control *Prss56*^*Cre/+*^;*R26*^*tdTomato/+*^ retina. Of note, while tdTomato expression is enriched in the peripheral region of the retina in control *Prss56*^*Cre/+*^;*R26*^*tdTomato/+*^ eyes, tdTomato distribution is more uniform in *Prss56* mutant retina (*Prss56*^*Cre/glcr4*^). (**D**) Representative images of retinal sections showing absence of immunolabeling when using rabbit or goat IgG isotypes as negative controls. CB, ciliary body; GCL, ganglionic cell layer; INL, inner nuclear layer; ONL, outer nuclear layer; P, postnatal day. Scale bars; 500 μm (**A**), 100 μm for P10 and 50 μm for P15 (**B**), and 50 μm (**C**).
(TIF)

S4 Fig Generation of conditional *Prss56* mutant mice. (**A**) *Prss56* alleles and targeting construct. Top: Wild-type *Prss56* allele (exons indicated as solid black rectangles). Middle: Targeting vector containing 2 LoxP sites (black triangles) flanking *Prss56* exons 3 and 4 and a Neomycin selection cassette (Neo) flanked by 2 Frt sites (green triangles). Insertion of the targeting vector by homologous recombination in embryonic stem (ES) cells yielded *Prss56*^*F_Neo*^. *Prss56*^*F_Neo*^ ES cells were used to generate chimeric mice that were bred to mice expressing flippase for excision of the Neomycin selection cassette to generate mice carrying the conditional *Prss56* mutant allele (*Prss56*^*F*^*)*. The *Prss56*^*F*^ allele expresses normally and behaves as the wild-type *Prss56* allele in the absence of Cre recombinase activity. Bottom: Cre recombinase activation causes deletion of exons 3 and 4 resulting in a frameshift mutation and premature stop codon, rendering the *Prss56* gene inactive. (**B**-**C**) *Prss56* gene targeting was confirmed by Southern hybridization (not shown) and PCR. PCR analyses using various primer combinations (gray triangles in **A**) are shown. PCR amplification of DNA from wild-type mice (lane 1), mice heterozygous or homozygous for the *Prss56* conditional allele (*Prss56*^*F/+*^, lane 2; and *Prss56*^*F/F*^, lane 3), and mice homozygous for the *Prss56* conditional allele in presence of a ubiquitous Sox2-Cre recombinase (lane 4). PCR reactions using the F1R1 primer pair gives a product that is about 34 bp longer in mice carrying the *Prss56*^*F*^ allele compared to wild-type mice. In the presence of Cre recombinase, deletion of exon 3 and 4 from the *Prss56*^*F*^ allele gives no PCR product. PCR reactions using the F2R2 primer pair lead to product sizes of 280bp for the wild-type allele and 350bp for the *Prss56*^*F*^ allele and no PCR product for the *Prss56*^*F*^ allele following Cre activation. PCR reactions using the primer pair F1R2 give rise to product sizes of 820bp and 920bp for the wild-type and *Prss56*^*F*^ alleles, respectively. Additionally, primer pair F1R2 confirmed excision of exons 3 and 4 from the *Prss56*^*F*^ allele following CRE activation as shown by the presence of a shorter PCR product size of 290bp.
(TIF)

S5 Fig Ubiquitous Sox2-Cre-mediated conditional ablation of *Prss56* recapitulates the ocular phenotype observed *in Prss56*^*-/-*^ mice. (**A**) *Prss56*^*F/F*^ mice were bred to mice ubiquitously expressing Cre recombinase under the control of the *Sox2* promoter (Sox2-Cre). Representative images of slit lamp examination by broad-beam illumination of *Prss56*^*F/+*^*;Sox2-Cre* and *Prss56*^*F/F*^*;Sox2-Cre* to assess ocular structures including the iris, pupil, and lens at 2 months of age. *Prss56*^*F/F*^*;Sox2-Cre* eyes were indistinguishable from control *Prss56*^*F/+*^*;Sox2-Cre* eyes and did not exhibit any obvious structural abnormalities. (**B, C**) *Prss56*^*F/F*^;*Sox2-Cre* eyes exhibit a significant reduction in axial length (**B**) and equatorial diameter (**C**) compared to control *Prss56*^*F/+*^*;Sox2-Cre* eyes. Values are presented as mean ± SD; * p<0.05, ** p<0.01, *** p<0.001, t-test. N≥ 4 per genotype.
(TIF)

S6 Fig Increase in ocular anterior chamber depth following *Prss56* ablation at P18. (**A**) Ocular biometry following tamoxifen injection at P18 shows that lens diameter is indistinguishable between *Prss56*^*F/F*^*;Ubc-Cre*^*ERT2*^ and control *Prss56*^*F/+*^*;Ubc-Cre*^*ERT2*^ mice. (**B**) *Prss56*^*F/F*^*;Ubc-Cre*^*ERT2*^ mice display a slight increase in ACD compared to the control group. Values are presented as mean ± SD. For comparison between mutant and control eyes, * p<0.05, **p<0.01, *** p<0.001, t-test.
(TIF)

S7 Fig The ocular dimensions of *Egr1*^*+/-*^*;Prss56*^*+/-*^ mice are indistinguishable from that of *Egr1*^*+/+*^*;Prss56*^*+/+*^. (**A**-**D**) Histograms showing that ocular axial length (**A**), retinal thickness (**B**), anterior chamber depth (ACD), vitreous chamber depth (VCD) (**C**), and lens thickness (**D**) are indistinguishable in *Egr1*^***+/-***^*;Prss56*^*+/-*^ and *Egr1*^***+/+***^*;Prss56*^*+/+*^ mice at P30. Values are presented as mean ± SD.
(TIF)

S1 Table Summary of ocular measurements in *Prss56* mutant mice across ages. (DOCX)

S2 Table Summary of ocular measurements in *Prss56* conditional mutant mice. (DOCX)

S3 Table Summary of ocular measurements of *Prss56/Egr1* genetic interaction study. (DOCX)

S4 Table List of genotyping primers. (DOCX)

S5 Table List of qPCR primers. (DOCX)
