# Supplementary figures and images for: Müller glia-derived PRSS56 is required to sustain ocular axial growth and prevent refractive error
Source: PLoS Genet. 2018 Mar 12;14(3):e1007244. doi: 10.1371/journal.pgen.1007244 (PMC5864079; doi:10.1371/journal.pgen.1007244)

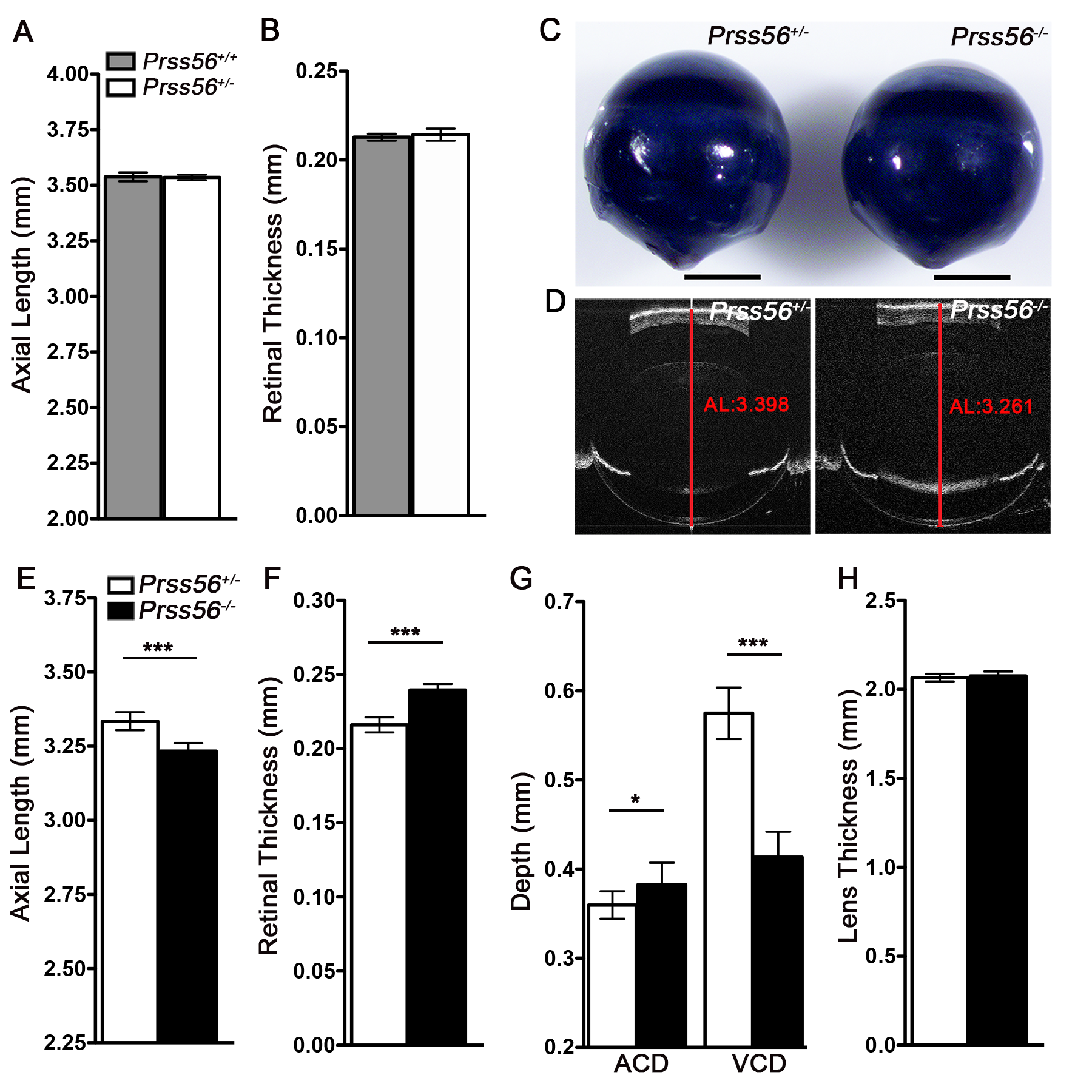

Supplement: S1 Fig — (A, B) Histograms showing that ocular axial length (A) and retinal thickness (B) are indistinguishable in Prss56+/- and Prss56+/+ mice at 3 months of age. (C) Representative images of enucleated eyes showing a modestly reduced size in Prss56-/- compared to Prss56+/- mice (shown are P15 eyes). (D) Representative optical coherence tomography images of 2 months old eyes. (E-H) Histograms showing reduced ocular axial length (E), increased retinal thickness (F), increased anterior chamber depth (ACD), decreased vitreous chamber depth (VCD) (G) in Prss56-/- eyes compared to Prss56+/- mice at 2 months of age. (H) Lens thickness was indistinguishable between Prss56-/- and Prss56+/- eyes. Data are presented as mean ± SD, ***p<0.001, t-test. In (A) N = 6 and 4 for Prss56+/- and Prss56+/+, respectively; in (E-H) N = 10 and 9 for Prss56+/- and Prss56-/-, respectively. (TIF) [file pgen.1007244.s001.tif]

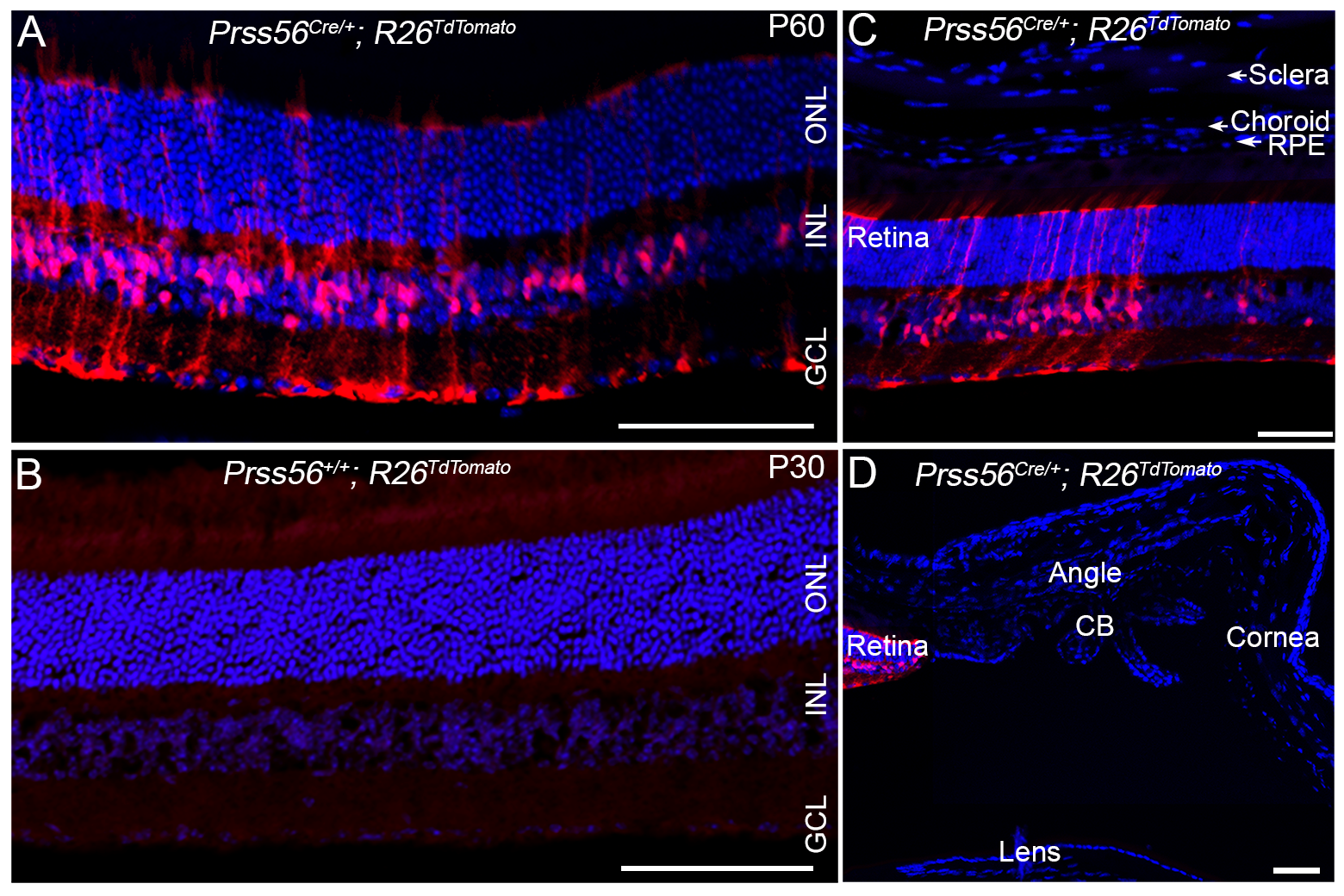

Supplement: S2 Fig — (A-D) Representative images of Prss56Cre/+;R26tdTomato/+ and Prss56+/+;R26tdTomato/+ ocular sections showing that tdTomato labeling (in red, reporting Prss56 expression) is restricted to the retina in Prss56Cre/+;R26tdTomato/+ mice. (B) tdTomato was not detected in the absence of Cre expression (Prss56+/+;R26tdTomato/+). tdTomato-labeled cells were enriched in the peripheral region and relatively sparser in the central region of the retina (A, C, the peripheral and central regions of the retina are oriented left to right). (C, D) tdTomato expression was not detected in the iridocorneal angle, ciliary body (CB), cornea, lens, sclera, choroid or retinal pigment epithelium (RPE). Scale bars = 100μm. (TIF) [file pgen.1007244.s002.tif]

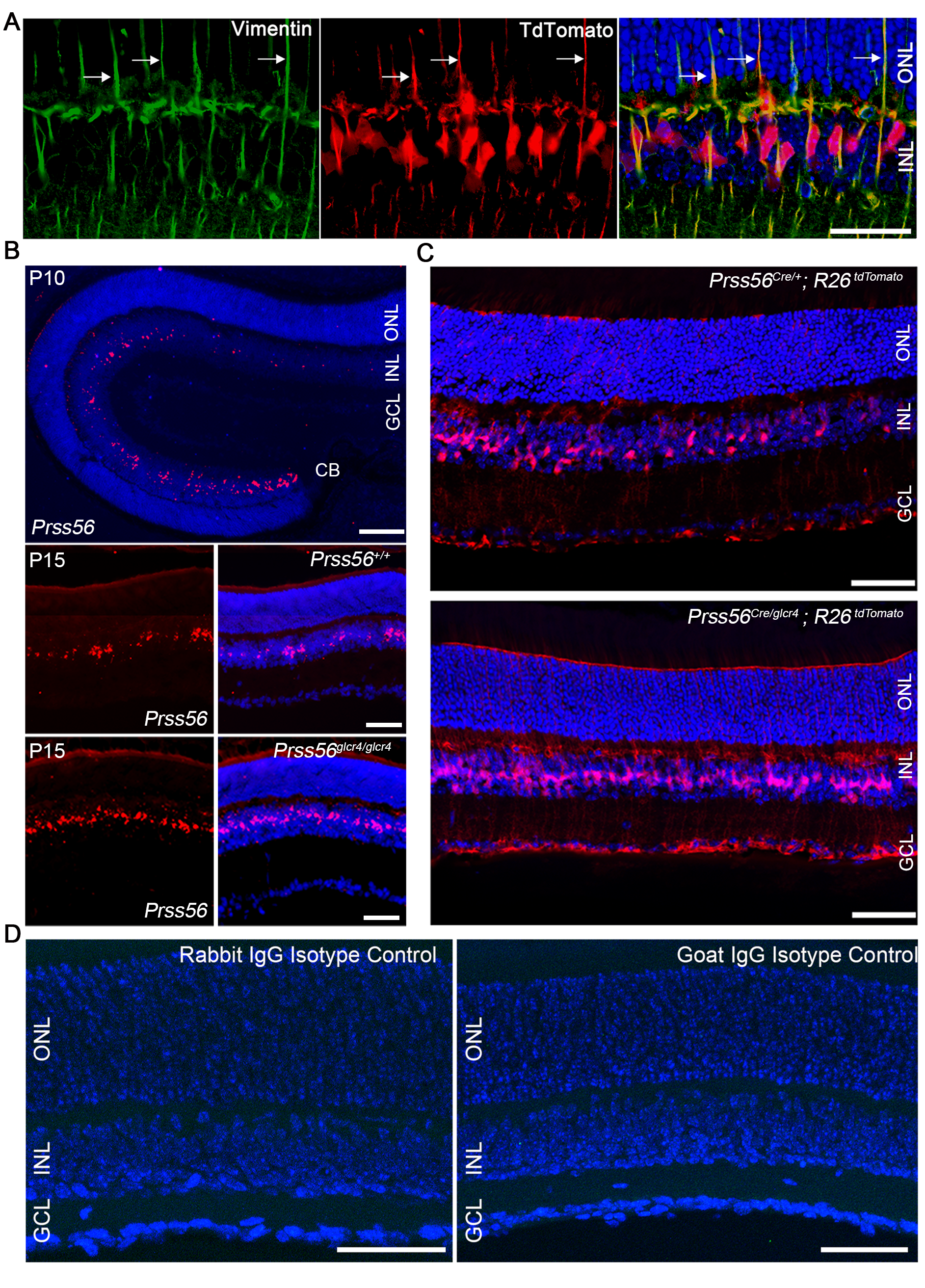

Supplement: S3 Fig — (A) Representative image of P20 Prss56Cre/+;R26tdTomato/+ retinal sections immunolabeled for the glial cell marker vimentin, showing colocalization of tdTomato and vimentin (arrows). (B) Detection of Prss56 mRNA expression at P10 and P15 using QuantiGene View RNA in situ hybridization. Top panel: A representative image of P10 retina showing Prss56 expression predominantly in the peripheral region of the retina. At P15, increased Prss56 expression was detected in the inner nuclear layer of the retina in mutant mice compared to their wild-type littermates. (C) Representative P30 eye sections showing an increased number of tdTomato positive cells in a mutant Prss56Cre/glcr4;R26tdTomato/+ retina compared to control Prss56Cre/+;R26tdTomato/+ retina. Of note, while tdTomato expression is enriched in the peripheral region of the retina in control Prss56Cre/+;R26tdTomato/+ eyes, tdTomato distribution is more uniform in Prss56 mutant retina (Prss56Cre/glcr4). (D) Representative images of retinal sections showing absence of immunolabeling when using rabbit or goat IgG isotypes as negative controls. CB, ciliary body; GCL, ganglionic cell layer; INL, inner nuclear layer; ONL, outer nuclear layer; P, postnatal day. Scale bars; 500 μm (A), 100 μm for P10 and 50 μm for P15 (B), and 50 μm (C). (TIF) [file pgen.1007244.s003.tif]

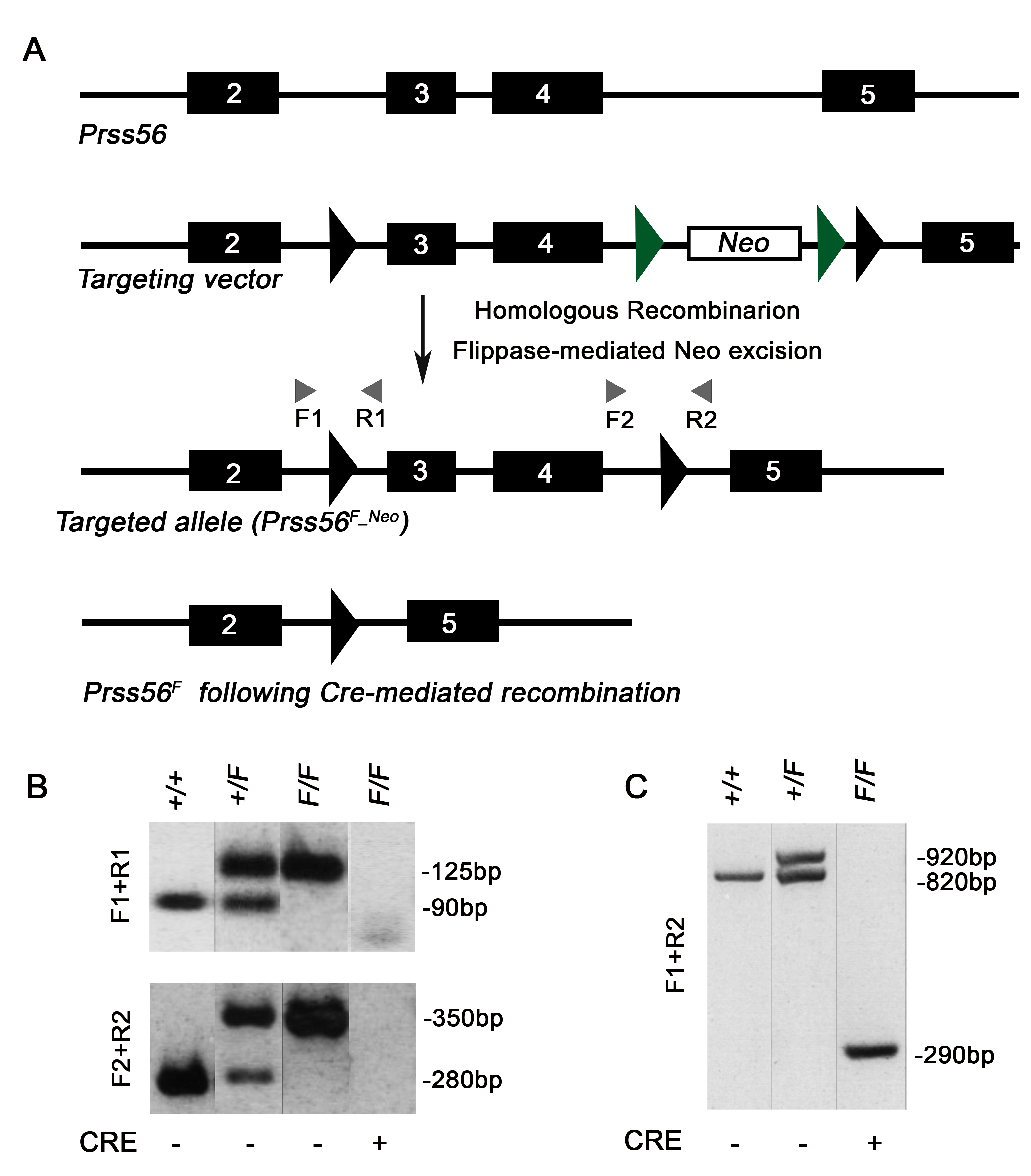

Supplement: S4 Fig — (A) Prss56 alleles and targeting construct. Top: Wild-type Prss56 allele (exons indicated as solid black rectangles). Middle: Targeting vector containing 2 LoxP sites (black triangles) flanking Prss56 exons 3 and 4 and a Neomycin selection cassette (Neo) flanked by 2 Frt sites (green triangles). Insertion of the targeting vector by homologous recombination in embryonic stem (ES) cells yielded Prss56F_Neo. Prss56F_Neo ES cells were used to generate chimeric mice that were bred to mice expressing flippase for excision of the Neomycin selection cassette to generate mice carrying the conditional Prss56 mutant allele (Prss56F). The Prss56F allele expresses normally and behaves as the wild-type Prss56 allele in the absence of Cre recombinase activity. Bottom: Cre recombinase activation causes deletion of exons 3 and 4 resulting in a frameshift mutation and premature stop codon, rendering the Prss56 gene inactive. (B-C) Prss56 gene targeting was confirmed by Southern hybridization (not shown) and PCR. PCR analyses using various primer combinations (gray triangles in A) are shown. PCR amplification of DNA from wild-type mice (lane 1), mice heterozygous or homozygous for the Prss56 conditional allele (Prss56F/+, lane 2; and Prss56F/F, lane 3), and mice homozygous for the Prss56 conditional allele in presence of a ubiquitous Sox2-Cre recombinase (lane 4). PCR reactions using the F1R1 primer pair gives a product that is about 34 bp longer in mice carrying the Prss56F allele compared to wild-type mice. In the presence of Cre recombinase, deletion of exon 3 and 4 from the Prss56F allele gives no PCR product. PCR reactions using the F2R2 primer pair lead to product sizes of 280bp for the wild-type allele and 350bp for the Prss56F allele and no PCR product for the Prss56F allele following Cre activation. PCR reactions using the primer pair F1R2 give rise to product sizes of 820bp and 920bp for the wild-type and Prss56F alleles, respectively. Additionally, primer pair F1R2 confirme [file pgen.1007244.s004.tif]

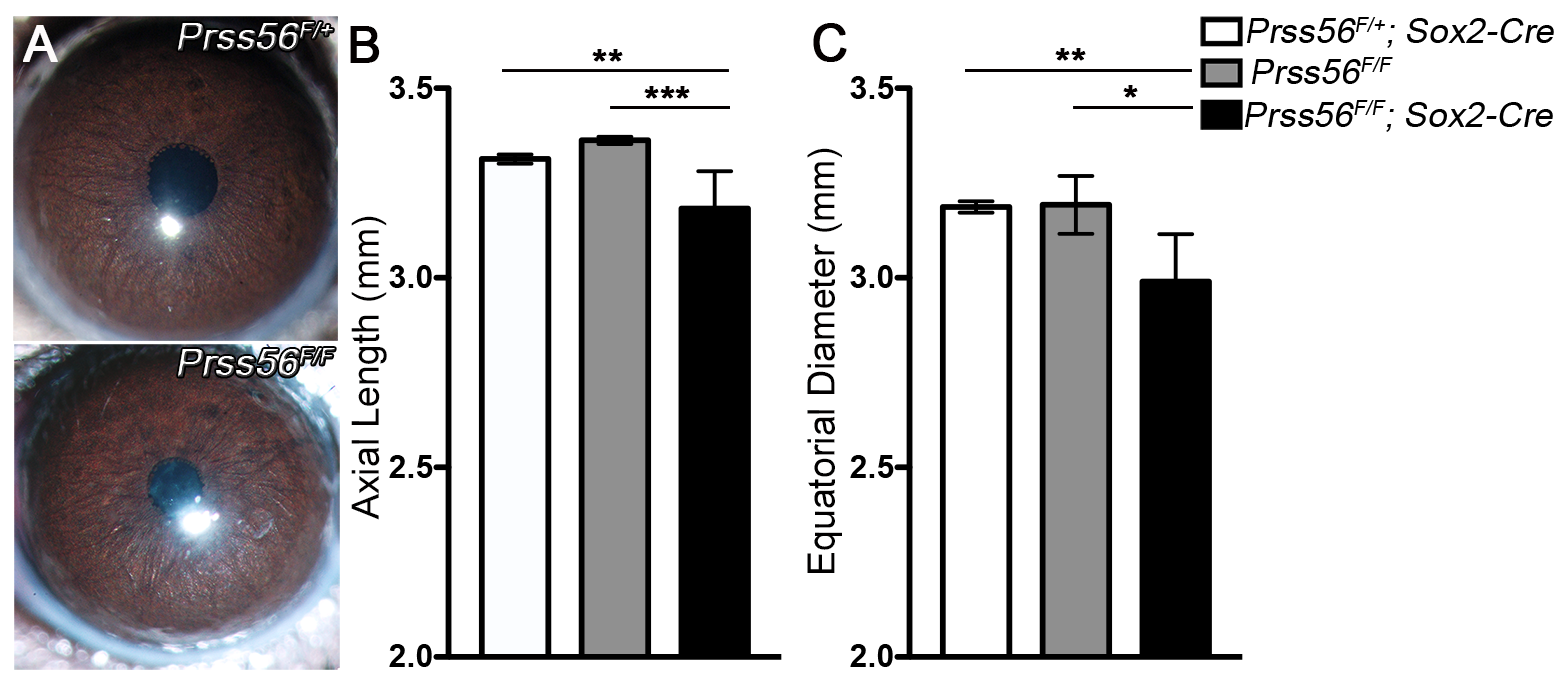

Supplement: S5 Fig — (A) Prss56F/F mice were bred to mice ubiquitously expressing Cre recombinase under the control of the Sox2 promoter (Sox2-Cre). Representative images of slit lamp examination by broad-beam illumination of Prss56F/+;Sox2-Cre and Prss56F/F;Sox2-Cre to assess ocular structures including the iris, pupil, and lens at 2 months of age. Prss56F/F;Sox2-Cre eyes were indistinguishable from control Prss56F/+;Sox2-Cre eyes and did not exhibit any obvious structural abnormalities. (B, C) Prss56F/F;Sox2-Cre eyes exhibit a significant reduction in axial length (B) and equatorial diameter (C) compared to control Prss56F/+;Sox2-Cre eyes. Values are presented as mean ± SD; * p<0.05, ** p<0.01, *** p<0.001, t-test. N≥ 4 per genotype. (TIF) [file pgen.1007244.s005.tif]

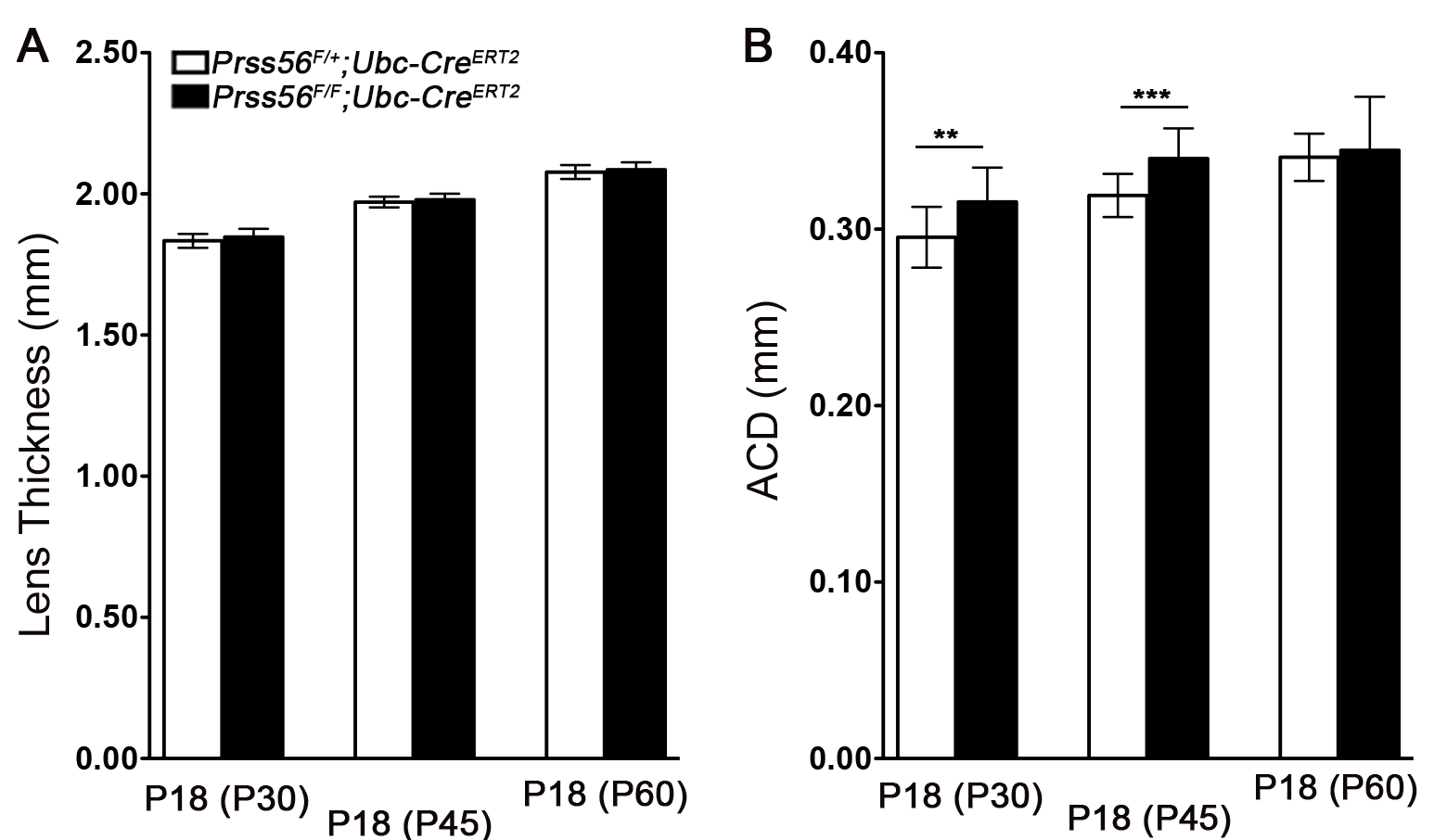

Supplement: S6 Fig — (A) Ocular biometry following tamoxifen injection at P18 shows that lens diameter is indistinguishable between Prss56F/F;Ubc-CreERT2 and control Prss56F/+;Ubc-CreERT2 mice. (B) Prss56F/F;Ubc-CreERT2 mice display a slight increase in ACD compared to the control group. Values are presented as mean ± SD. For comparison between mutant and control eyes, * p<0.05, **p<0.01, *** p<0.001, t-test. (TIF) [file pgen.1007244.s006.tif]

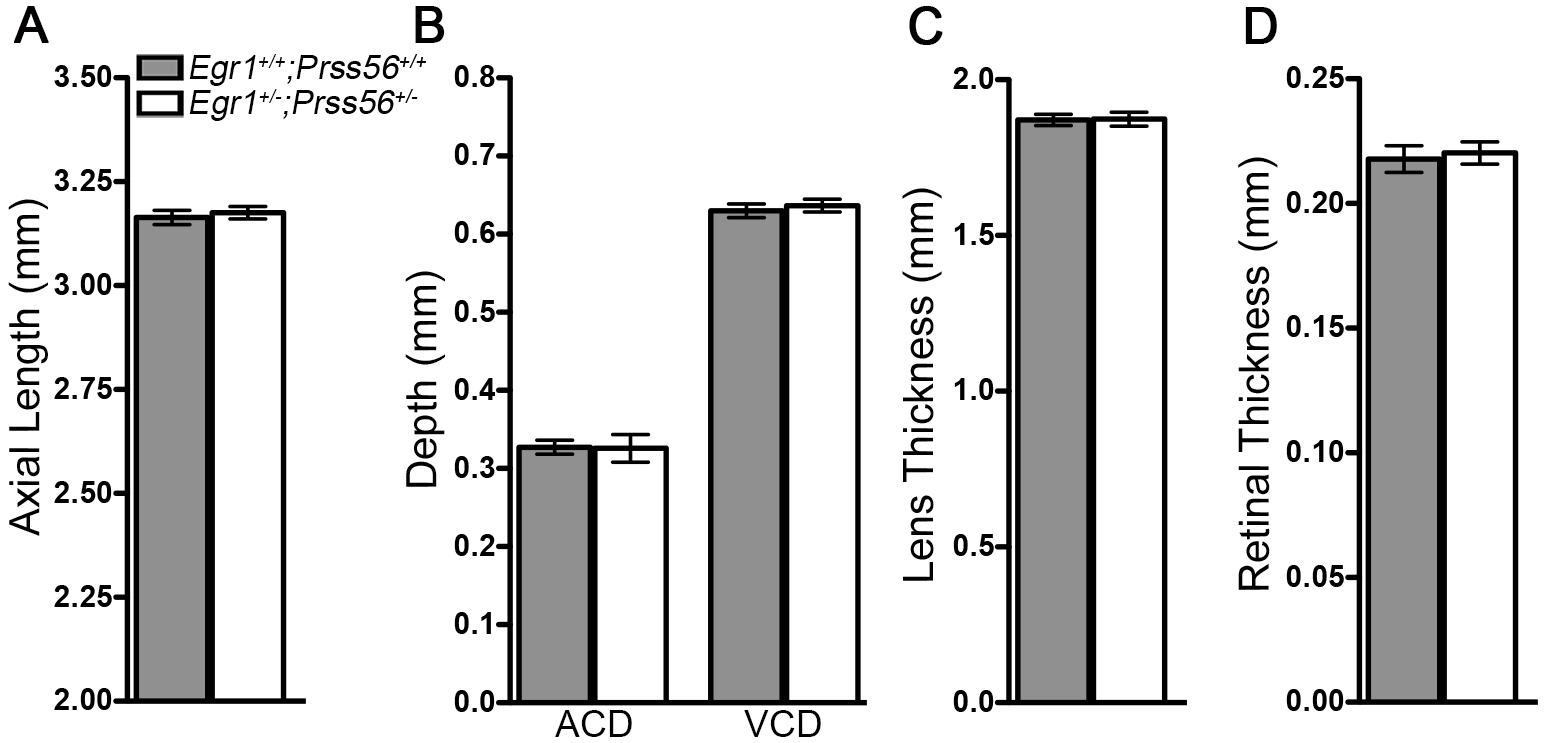

Supplement: S7 Fig — (A-D) Histograms showing that ocular axial length (A), retinal thickness (B), anterior chamber depth (ACD), vitreous chamber depth (VCD) (C), and lens thickness (D) are indistinguishable in Egr1+/-;Prss56+/- and Egr1+/+;Prss56+/+ mice at P30. Values are presented as mean ± SD. (TIF) [file pgen.1007244.s007.tif]
