# Supplementary material for: Müller glia-derived PRSS56 is required to sustain ocular axial growth and prevent refractive error
Source: PLoS Genet. 2018 Mar 12;14(3):e1007244. doi: 10.1371/journal.pgen.1007244 (PMC5864079; doi:10.1371/journal.pgen.1007244)
Supplement: S1 Table — (DOCX) [file pgen.1007244.s008.docx]

**Table S1. Summary of ocular measurements in *Prss56* mutant mice across ages**

| Genotype | Age | Number of eyes | Weight  (g) | Axial Length (μm) | VCD  (μm) | Retinal Thickness  (μm) | |
| --- | --- | --- | --- | --- | --- | --- | --- |
| *Prss56^glcr4/+^* | P5 | 10 | 2.88 ± 0.18 | 2203 ± 33 | N/D | 252.3 ± 1 | |
| *Prss56^glcr4/glcr4^* | P5 | 11 | 2.75 ± 0.23 | 2159 ± 49 | N/D | 264.9 ± 7 | |
| *Prss56^glcr4/+^* | P6 | 12 | 3.43 ± 0.24 | 2336 ± 51 | N/D | | 231 ± 6 |
| *Prss56^glcr4/glcr4^* | P6 | 18 | 3.33 ± 0.083 | 2260 ± 48 | N/D | | 243.1 ± 8 |
| *Prss56^glcr4/+^* | P8 | 6 | 4.57 ± 0.21 | 2583 ± 43 | N/D | | 214.8 ± 2 |
| *Prss56^glcr4/glcr4^* | P8 | 6 | 4.42 ± 0.11 | 2519 ± 16 | N/D | | 226.7 ± 2 |
| *Prss56^Cre/+^* | P15 | 18 | N/D | 2858 ± 87 | N/D | | N/D |
| *Prss56^Cre/Cre^* | P15 | 16 | N/D | 2721 ± 64 | N/D | | N/D |
| *Prss56^glcr4/+^* | P17 | 6 | 7.2 ± 0.65 | 2969 ± 43 | 670.5 ± 31 | | 235 ± 2 |
| *Prss56^glcr4/glcr4^* | P17 | 6 | 8.2 ± 0.37 | 2889 ± 33 | 560.8 ± 51 | | 254.5 ± 4 |
| *Prss56^Cre/+^* | P25 | 10 | N/D | 3091 ± 74 | N/D | | N/D |
| *Prss56^Cre/Cre^* | P25 | 12 | N/D | 2954 ± 72 | N/D | | N/D |
| *Prss56^Cre/+^* | 1M | 4 | 15.8 ± 0.28 | 3181 ± 20 | 609.3 ± 12 | | 217.5 ± 4 |
| *Prss56^Cre/Cre^* | 1M | 6 | 17.1 ± 0.87 | 3115 ± 23 | 469.8 ± 12 | | 240.8 ± 2 |
| *Prss56^Cre/+^* | 2M | 10 | 20.45 ± 2.42 | 3344± 30 | 574.8 ± 29 | | 216.1 ± 5 |
| *Prss56^Cre/Cre^* | 2M | 9 | 21.54 ± 2.95 | 3233 ± 19 | 413.4 ± 41 | | 239.5 ± 4 |
