# Supplementary material for: Müller glia-derived PRSS56 is required to sustain ocular axial growth and prevent refractive error
Source: PLoS Genet. 2018 Mar 12;14(3):e1007244. doi: 10.1371/journal.pgen.1007244 (PMC5864079; doi:10.1371/journal.pgen.1007244)
Supplement: S2 Table — (DOCX) [file pgen.1007244.s009.docx]

**Table S2. Summary of ocular measurements in *Prss56* conditional mutant mice**

| Genotype | Tamoxifen administration day | OCT measurement day | Number of eyes | Weight (g) | | Axial Length (μm) | VCD  (μm) | Retinal Thickness  (μm) |
| --- | --- | --- | --- | --- | --- | --- | --- | --- |
| *Rax-Cre;Prss56^F/+^* | Untreated | P17 | 6 | 7.52 ± 0.84 | | 2866 ± 17 | 684 ± 23 | 234.3 ± 4 |
| *Rax-Cre;Prss56^F/F^* | Untreated | P17 | 6 | 9.23 ± 0.63 | | 2917 ± 42 | 676.5 ± 12 | 238.2 ± 3 |
| *+/+; Prss56^F/F^* | Treated (P8) | P17 | 8 | 7.125 ± 0.48 | | 2867 ± 24 | N/D | 233 ± 6 |
| *Rax-Cre;Prss56^F/+^* | Treated (P8) | P17 | 6 | 7.97 ± 0.9 | | 2869 ± 16 | 723 ± 71 | 230 ± 7 |
| *Rax-Cre;Prss56^F/F^* | Treated (P8) | P17 | 6 | 6.15 ± 0.77 | | 2725 ± 37 | 537.3 ± 74 | 269.2 ± 8 |
| *Ubc-Cre;Prss56^F/+^* | Untreated | P17 | 8 | 7.325 ± 0.8 | | 2910 ± 22 | 633 ± 44 | 237.9 ± 5 |
| *Ubc-Cre;Prss56^F/F^* | Untreated | P17 | 6 | 7.27 ± 0.63 | | 2891 ± 29 | 627.8 ± 11 | 238 ± 2 |
| *Ubc-Cre;Prss56^F/+^* | Treated (P6) | P17 | 6 | 7.13 ± 0.86 | | 2912 ± 22 | 630.7 ± 49 | 239.3 ± 3 |
| *Ubc-Cre;Prss56^F/F^* | Treated (P6) | P17 | 8 | 6.65 ± 1.64 | | 2762 ± 37 | 559.3 ± 18 | 263.6 ± 4 |
| *Ubc-Cre;Prss56^F/+^* | Treated (P8) | P17 | 8 | 7.33 ± 0.17 | | 2920 ± 18 | 630 ± 26 | 240.4 ± 3 |
| *Ubc-Cre;Prss56^F/F^* | Treated (P8) | P17 | 8 | 6.77 ± 0.45 | | 2844 ± 23 | 566 ± 39 | 255.8 ± 4 |
|  |  |  |  |  | |  |  |  |
| *Ubc-Cre;Prss56^F/F^* | Untreated | P30 | 5 | 15.5 ± 0.92 | | 3172 ± 17 | 630.2 ± 14 | 221.8 ± 1 |
| *Ubc-Cre;Prss56^F/+^* | Treated (P13) | P30 | 9 | 14.22 ± 0.96 | | 3158 ± 39 | 622.1 ± 16 | 221.1 ± 5 |
| *Ubc-Cre;Prss56^F/F^* | Treated (P13) | P30 | 7 | 14.73 ± 1.17 | | 3119 ± 11 | 575.7 ± 2 | 225.6 ± 5 |
| *Ubc-Cre;Prss56^F/F^* | Untreated | P45 | 8 | 19.18 ± 2.55 | | 3279 ± 27 | 600.3 ± 28 | 213.6 ± 5 |
| *Ubc-Cre;Prss56^F/+^* | Treated (P13) | P45 | 10 | 18.5 ± 2.52 | | 3293 ± 51 | 587.4 ± 15 | 215.5 ± 4 |
| *Ubc-Cre;Prss56^F/F^* | Treated (P13) | P45 | 8 | 19 ± 1.15 | | 3246 ± 31 | 541.9 ± 14 | 226 ± 7 |
|  |  |  |  |  | |  |  |  |
| *Ubc-Cre;Prss56^F/+^* | Treated (P18) | P30 | 14 | 13.84 ± 1.21 | | 3104 ± 39 | 622.5 ± 22 | 221.8 ± 4 |
| *Ubc-Cre;Prss56^F/F^* | Treated (P18) | P30 | 14 | 13.77 ± 1.20 | | 3090 ± 29 | 594.7 ± 18 | 225.5 ± 4 |
|  |  |  |  |  | |  |  |  |
| *Ubc-Cre;Prss56^F/+^* | Treated (P18) | P45 | 14 | 19.11± 1.96 | | 3233 ± 44 | 595.4 ± 19 | 217.5 ± 4 |
| *Ubc-Cre;Prss56^F/F^* | Treated (P18) | P45 | 14 | 18.64 ± 1.62 | | 3212 ± 35 | 547.6 ± 31 | 223.1 ± 5 |
|  |  |  |  |  | |  |  |  |
| *Ubc-Cre;Prss56^F/+^* | Treated (P18) | P60 | 13 | 20.61± 2.36 | | 3317 ± 41 | 565.9 ± 12 | 216.1 ± 4 |
| *Ubc-Cre;Prss56^F/F^* | Treated (P18) | P60 | 14 | 19.73 ± 1.63 | | 3288 ± 33 | 510 ± 36 | 224.1 ± 5 |
|  |  |  |  |  |  | |  |  |
