# Supplementary material for: Müller glia-derived PRSS56 is required to sustain ocular axial growth and prevent refractive error
Source: PLoS Genet. 2018 Mar 12;14(3):e1007244. doi: 10.1371/journal.pgen.1007244 (PMC5864079; doi:10.1371/journal.pgen.1007244)
Supplement: S3 Table — (DOCX) [file pgen.1007244.s010.docx]

**Table S3. Summary of ocular measurements of *Prss56/Egr1* genetic interaction study**

| Genotype | OCT measurement day | Number of eyes | Weight (g) | | Axial Length (μm) | VCD  (μm) | Retinal Thickness  (μm) |
| --- | --- | --- | --- | --- | --- | --- | --- |
| *Prss56^+/-^; Egr1^+/-^* | P10 | 4 | 6.4 ± 0.14 | | 2714 ± 9 | 710 ± 23 | N/D |
| *Prss56^+/-^; Eg1^-/-^* | P10 | 6 | 6.27 ± 0.4 | | 2802 ± 31 | 788 ± 10 | N/D |
| *Prss56^-/-^; Egr1^+/-^* | P10 | 7 | 6.2 ± 0.18 | | 2667 ± 29 | 642.3 ± 32 | N/D |
| *Prss56^-/-^; Egr1^-/-^* | P10 | 6 | 5.9 ± 0.2 | | 2749 ± 77 | 758.2 ± 46 | N/D |
| *Prss56^+/-^; Egr1^+/-^* | P14 | 12 | 8.23 ± 0.5 | | 2848 ± 31 | 664.8 ± 28 | N/D |
| *Prss56^+/-^; Eg1^-/-^* | P14 | 6 | 7.67 ± 0.06 | | 2900 ± 20 | 740.2 ± 30 | N/D |
| *Prss56^-/-^; Egr1^+/-^* | P14 | 6 | 7.7 ± 1.01 | | 2765 ± 46 | 563 ± 21 | N/D |
| *Prss56^-/-^; Egr1^-/-^* | P14 | 6 | 7.43 ± 0.72 | | 2847 ± 43 | 686.5 ± 51 | N/D |
| *Prss56^+/-^; Egr1^+/-^* | P30 | 12 | 16.07 ± 1.45 | | 3161 ± 29 | 632.8 ± 22 | 214.7 ± 4 |
| *Prss56^+/-^; Eg1^-/-^* | P30 | 8 | 15 ± 0.91 | | 3205 ± 25 | 682.4 ± 24 | 219.1 ± 4 |
| *Prss56^-/-^; Egr1^+/-^* | P30 | 12 | 13.61 ± 1.45 | | 3076 ± 23 | 518.1 ± 35 | 241.2 ± 5 |
|  |  |  |  | |  |  |  |
| *Prss56^-/-^; Egr1^-/-^* | P30 | 7 | 15.38 ± 0.93 | | 3174 ± 28 | 599.1 ± 56 | 230.1 ± 7 |
| *Prss56^+/-^; Egr1^+/-^* | P60 | 10 | 18.62 ± 1.17 | | 3342 ± 25 | N/D | 214.6. ± 3 |
| *Prss56^+/-^; Eg1^-/-^* | P60 | 11 | 20.67 ± 1.75 | | 3467 ± 49 | N/D | 213.7 ±5 |
| *Prss56^-/-^; Egr1^+/-^* | P60 | 12 | 21.57 ± 1.51 | | 3249 ± 33 | N/D | 243.2 ± 5 |
| *Prss56^-/-^; Egr1^-/-^* | P60 | 13 | 19.91± 1.95 | 3322 ± 44 | | N/D | 235.6 ± 1 |
|  |  |  |  |  | |  |  |
