# Supplementary material for: Müller glia-derived PRSS56 is required to sustain ocular axial growth and prevent refractive error
Source: PLoS Genet. 2018 Mar 12;14(3):e1007244. doi: 10.1371/journal.pgen.1007244 (PMC5864079; doi:10.1371/journal.pgen.1007244)
Supplement: S4 Table — (DOCX) [file pgen.1007244.s011.docx]

**Table S4. List of genotyping primers**

| Allele (mouse strain) | Primer name | Primer sequence |
| --- | --- | --- |
| *Prss56^glcr4^*  (C57BL/6.Cg-Prss56 glcr4/SjJ) | Prss56 glcr4 F1 | 5' TGGCTCCAGAAACCAAAGCCGGAA  GAGCGCCCGGAAACAAAGAGT 3' |
|  | Prss56 glcr4 F2 | 5' GCGGCGCCCGGAAACAAAAGGA 3' |
|  | Prss56 glcr4 R | 5' TCCTGGAAGAGAGGGAGTGA 3' |
| *Prss56^Cre^* | Prss56 Cre F | 5’ CAG GGC ATC GTT TCC CTG AG 3’ |
| (C57Bl/6.Cg-Prss56tm) | Prss56 Cre R WT | 5’ GAC AGG CGC GTG TAC AGT GG 3’ |
|  | Prss56 Cre R Cre | 5’ CCA TGA GTG AAC GAA CCT GG 3’ |
| *Prss56^F^* | Prss56 cKO F1 | 5' CTCAGGTGGTCACAGTTCAT 3 |
| (C57BL/6.Cg-Prss56tm1/SjJ ) | Prss56 cKO R1 | 5' TGGGCCTCATCTTTCAACC 3' |
|  | Prss56 cKO R3 | 5' CCATCCAGTGAGCCTGTAAG 3' |
| *R26^tDTomato^* | Tom1 | 5’ AAG GGA GCT GCA GTG GAG TA 3’ |
| (Gt(ROSA)26Sortm14(CAG-tdTomato)Hze) | Tom2 | 5’ CCG AAA ATC TGT GGG AAG TC 3’ |
|  | Tom3 | 5’ GGC ATT AAA GCA GCG TAT CC 3’ |
|  | Tom4 | 5’ CTG TTC CTG TAC GGC ATG G 3’ |
|  |  |  |
| *Rax-Cre* | Rax F | 5’ CCC TGA GGC TAA ACT TGC AG 3’ |
| (Raxtm1.1(cre/ERT2)Sbls/J) | Rax WT R | 5’ AGG TGT CTA GGA TGC CGT CT 3’ |
|  | Rax MUT R | 5’ AGG CAA ATT TTG GTG TAC GG 3’ |
| *Sox2-Cre* | Cr1 | 5’ TGA TGA GGT TCG CAA GAA CC 3’ |
| (Sox2-Cre:Tg(Sox2-cre)1Amc/J) | Cr2 | 5’ CCA TGA GTG AAC GAA CCT GG 3’ |
|  |  |  |
| *Ubc-Cre* | Cr1 | 5’ TGA TGA GGT TCG CAA GAA CC 3’ |
| (C57Bl/6.Cg-Tg(UBC-Cre/ERT2)1Ejb) | Cr2 | 5’ CCA TGA GTG AAC GAA CCT GG 3’ |
