# Supplementary material for: Müller glia-derived PRSS56 is required to sustain ocular axial growth and prevent refractive error
Source: PLoS Genet. 2018 Mar 12;14(3):e1007244. doi: 10.1371/journal.pgen.1007244 (PMC5864079; doi:10.1371/journal.pgen.1007244)
Supplement: S5 Table — (DOCX) [file pgen.1007244.s012.docx]

**Table S5. List of qPCR primers**

| Gene | Forward Primer | Reverse Primer |
| --- | --- | --- |
| *House Keeping Genes* |  |  |
| *Actb* | 5' CCCTGAGGAGCACCCTGTGC 3' | 5' GGCTGGGGTGTTGAAGGTCT 3' |
| *Hprt1* | 5' TGCCGAGGATTTGGAAAAAGTGT 3' | 5' GTGATGGCCTCCCATCTCCT 3' |
| *Mapk1* | 5' TTGAACAGGCTCTGGCCCAC 3' | 5' TGAATGGCGCTTCAGCAATGG 3' |
| *Genes of Interest* |  |  |
| *Prss56* | 5' ACCTGGACGCCCTAGACCTC 3' | 5' TGTTGGCAACGCCTTGATGT 3' |
